# Toward Industry 5.0: A WebSocket–S7 Bridge for Low-Latency, IEC 61588-Compliant Digital Twins in Remote Industrial Automation

**DOI:** 10.1371/journal.pone.0342004

**Published:** 2026-05-11

**Authors:** Mohammed Hlayel, Hairulnizam Mahdin, Mohammad Hayajneh, Heru Nurwarsito

**Affiliations:** 1 Fatima College of Health Sciences, Institute of Applied Technology, Abu Dhabi, UAE; 2 Faculty of Computer Science and Information Technology, Universiti Tun Hussein Onn Malaysia, Parit Raja, Johor, Malaysia; 3 Faculty of Computer Science, University of Brawijaya, Malang, Indonesia; 4 College of Information Technology, United Arab Emirates University, Al-Ain, Abu Dhabi, UAE; Northwestern Polytechnical University School of Software and Microelectronics, CHINA

## Abstract

While Industry 4.0 established connectivity and automation as foundations of smart factories, the emerging vision of Industry 5.0 emphasizes human-centric, resilient, and sustainable systems. Within this evolution, integrating Digital Twins (DTs) with Programmable Logic Controllers (PLCs) in cloud environments remains constrained by the lack of communication protocols that satisfy strict real-time requirements. To address this gap, this paper introduces and validates a WebSocket–S7 protocol bridge for low-latency, full-duplex communication between Siemens S7 PLCs and cloud-hosted DT models. The main contribution of this work is the design and experimental validation of a lightweight, event-driven WebSocket–S7 architecture. This architecture decouples real-time PLC execution from cloud-based digital twin interaction, enabling deterministic performance over wide-area networks without relying on brokers or polling mechanisms. A comprehensive evaluation compares the proposed bridge with MQTT, OPC UA, and Modbus for real-time PLC–DT interactions managed through Node-RED. Using AWS as the cloud backend, the study assesses performance, scalability, and stability under single-user and multi-user emulated loads. Protocols were profiled using JMeter (load and latency), Wireshark (packet traces), and the Unity Profiler (CPU/memory on Android), with timing judged against IEC 61588 (≤100ms). In remote cloud deployment, WebSocket–S7 achieved an average round-trip time (RTT) of 87 ms, remaining within the IEC 61588 threshold. It outperformed alternative protocols by clear margins—34.6% lower RTT than MQTT (133 ms), 46.3% lower than OPC UA (162 ms), and 55.2% lower than Modbus (194 ms), yielding absolute gains of 46, 75, and 107 ms, respectively. Under multi-user emulation, WebSocket–S7 maintained sub-100 ms average response times for up to 40 concurrent users with near-zero error rates, while MQTT offered a balanced option for moderate-throughput scenarios. These trends reflect WebSocket’s lightweight framing and persistent, broker-less transport. Together, these characteristics minimize connection overhead and jitter in WAN conditions. The findings position WebSocket–S7 as a lightweight and scalable solution for remote DT–PLC integration, particularly when standardized protocols incur higher overhead. By consistently meeting IEC-compliant timing requirements, the proposed bridge provides a practical and robust foundation for deploying real-time digital twins in both educational and industrial automation settings.

## 1. Introduction

The convergence of Information and Communication Technologies (ICT) with industrial systems marks the advent of Industry 4.0, characterized by smart factories where sensors, machinery, and production systems communicate seamlessly, process data, and make autonomous decisions [1–3]. This shift toward real-time operations, remote monitoring, and decentralized control has transformed manufacturing into a more adaptive and efficient model. It has also enabled integrated supply chains and data-driven decision-making [4,5]. However, these advances also reveal persistent challenges in data exchange and system integration within smart factories, underscoring the need for effective bridges between the physical and digital realms [6,7].

At the core of this transformation are Digital Twins (DTs)—virtual replicas of physical assets that enable real-time monitoring, analysis, optimization, and simulation of industrial processes [8,9]. ISO 23247 distinguishes three categories: the Digital Twin Prototype (DTP), used during design and testing; the Digital Twin Instance (DTI), representing a specific asset in operation; and the Digital Twin Aggregate (DTA), which integrates multiple twins for system-level analysis [1,10]. Together, these approaches support scenario simulation, predictive maintenance, and lifecycle optimization.

Despite their promise, DT deployments are frequently constrained by legacy infrastructure and communication limitations. Upgrading older production systems to support modern Industrial Communication Protocols (ICPs) presents both technical and financial challenges [11]. Effective integration of Programmable Logic Controllers (PLCs)—the backbone of industrial automation—with cloud-based DTs depends on protocols that guarantee low-latency, reliable, and scalable data exchange [12–14]. Achieving this requires overcoming significant challenges in optimizing industrial networks and real-time communication protocols, especially for remote DT deployments. This demand for low-latency exchange makes real-time communication crucial for DTs in industrial settings, where it ensures timely data flow. It is equally important in education, where it enables interactive learning and collaborative simulations [15].

From a control standpoint, the PLC evaluates inputs and drives outputs cyclically and is thus agnostic to whether field I/O is physical or simulated, provided signal semantics and timing are preserved. In a DTP setting, virtual sensors generate the same logical events that physical sensors would, while actuator commands are consumed by a plant model rather than a physical machine. The controller processes both identically when I/O addressing, scan/sampling periods, and interface latency and jitter match the target deployment. This principle makes DTPs powerful tools for education. They are increasingly employed as cost-effective complements to physical labs, enabling engineering students to practice PLC programming and automation tasks in a safe and flexible environment. This aligns with the principles of Industry 5.0—human-centricity and resilience—where remote access to realistic industrial simulations reduces barriers to practical learning, ensures program continuity during disruptions (e.g., COVID-19), and supports inclusive, sustainable training at scale [16–19]. Remote and hybrid labs, which leverage simulations or hardware interfaces, further bridge the gap between theory and practice across manufacturing and complex systems [19–28].

Consistent with ISO 23247, a DTI mirrors a live asset during operation, whereas a DTP supports pre-deployment design and testing without a coupled physical counterpart [1]. Practically, this implies aligning simulation steps with the PLC scan, batching I/O updates, and bounding the end-to-end delay within the IEC 61588 requirement (below 100 ms for real-time operation), enabling effective testing and development even when a physical plant is unavailable [29].

Nonetheless, significant gaps persist in achieving real-time performance within remote DTP–PLC frameworks. Much of the existing research remains confined to local network environments [30,31], where latency and jitter are inherently lower and do not accurately represent the timing constraints of wide-area, cloud-mediated systems. Studies that extend to remote or hybrid configurations often report communication delays that exceed the thresholds required for deterministic, sub-100 ms interaction [32–34], undermining synchronization between virtual and physical assets. Furthermore, many implementations support only unidirectional data flow from PLCs to clients, limiting the closed-loop fidelity essential for interactive and adaptive twins.

This study addresses these gaps through a rigorous, multi-metric evaluation of communication protocols for real-time interactions between Siemens PLCs and cloud-hosted DTPs. Our central contribution is the design, implementation, and assessment of a novel WebSocket–S7 protocol bridge engineered to minimize latency and stabilize tail performance (e.g., 95th and 99th percentiles) across remote environments.

The novelty of the proposed WebSocket–S7 bridge lies in its hybrid architectural design and real-time performance engineering, which depart from standardized industrial communication models. While OPC UA, MQTT, and Modbus rely on middleware layers, brokers, or polling exchanges that introduce serialization and handshake overhead, the proposed bridge employs WebSocket solely as a lightweight, event-driven signaling interface. Native S7 read/write operations are executed locally within Node-RED, positioned near the PLC, while asynchronous WebSocket messages handle remote event triggers. This separation of concerns—WebSocket for signaling and Node-RED for execution—removes broker intermediation and redundant serialization. As a result, it enables deterministic, low-latency communication between cloud-based twins and physical PLCs. To meet stringent timing requirements, a custom real-time WebSocket library was developed, featuring optimized serialization, adaptive reconnection, and non-blocking I/O (Section 3.2.2). Together, these engineering optimizations enable the bridge to achieve IEC 61588 sub-100 ms compliance for real-time PLC–Digital Twin interaction across local and wide-area networks.

Building on this architecture, we hypothesize that WebSocket–S7 will outperform MQTT, OPC UA, and Modbus in latency, reliability, and scalability for high-frequency PLC–DT communication. Through a comprehensive experimental framework—including round-trip latency, throughput, server resource utilization, and client-side profiling—this study offers practical guidance for implementing robust, real-time DT solutions in both educational and industrial automation contexts, advancing the human-centric and resilient vision of Industry 5.0.

## 2. Background

Industrial communication protocols have been designed for many years to meet the real-time requirements of physical processes. With the emergence of Industry 4.0, there is an increasing emphasis on adopting ICT-based solutions that can serve as standardized, cross-industry communication methods. These approaches aim to ensure stable and low-latency data exchange. The rise of Internet-of-Things (IoT) platforms has introduced numerous protocols, each posing distinct challenges related to speed, bandwidth, scalability, and data integrity. Addressing these challenges requires a clear understanding of protocol architectures and their suitability for specific industrial contexts.

For this analysis, five representative protocols are examined: Siemens S7, OPC UA, Modbus, and MQTT—each widely implemented in industrial systems—together with WebSocket, evaluated here for its potential adaptation to real-time industrial communication [35–39]. This selection provides a comprehensive view of the communication landscape underpinning modern Digital Twin and Industry 4.0 environments.

### 2.1. Communication protocols

- *Siemens S7 Protocol (S7)* supports data exchange across industrial automation systems, including PLCs, HMIs, and SCADA platforms. It enables deterministic, real-time data transmission over Ethernet and serial connections and provides PUT/GET operations for efficient variable access. Favored for its robustness and reliability, S7 is natively integrated with Siemens SIMATIC PLCs, WinCC SCADA, and the TIA (Totally Integrated Automation) Portal suite [35].
- *WebSocket* establishes a full-duplex communication channel over a single TCP connection, reducing latency for real-time web applications such as online gaming and financial trading. Unlike HTTP, WebSocket enables continuous, bidirectional data exchange and integrates securely with modern web technologies via TLS/SSL encryption [39,40]. In industrial automation, WebSocket—initially designed for client–server communication—does not natively interface with PLCs. However, it performs effectively as a conduit between web-based clients and servers that manage PLC interactions. In this study, WebSocket serves as a pivotal link between Unity and Node-RED. It supports low-latency signaling essential for remote and dynamic industrial operations. This design enables the real-time integration of PLC data into web-based systems. As a result, it enhances monitoring, control, and visualization capabilities across distributed environments.
- *Modbus*, introduced by Modicon in 1979, remains one of the most widely used industrial communication protocols [37]. It supports both serial and Ethernet communication, with Modbus RTU for serial connections and Modbus TCP/IP for Ethernet networks. Its simple controller/agent architecture and lack of session overhead enable fast, straightforward data transactions [41]. A key strength of Modbus is its interoperability: as an open protocol, it enables communication among multi-vendor devices over Ethernet without proprietary constraints. In Modbus TCP, PLCs typically act as servers, responding to client requests encapsulated in TCP packets. These packets contain headers, device IDs, function codes, and variable status information.
- *OPC Unified Architecture (OPC UA)* is an open, platform-independent standard facilitating data exchange across information technology (IT) and operational technology (OT) domains. Evolving from earlier Microsoft-based implementations, OPC UA provides a secure, scalable, and service-oriented architecture for machine-to-machine communication. It forms the communication layer of the Reference Architecture Model for Industry 4.0 (RAMI 4.0) and is standardized under the IEC 62541 series [36]. Its server–client structure supports real-time data exchange, allowing clients to subscribe to variables hosted by servers. OPC UA’s integrated security, reliability, and extensibility make it a cornerstone protocol for Industry 4.0 deployments that require robust interoperability and structured data models.
- *Message Queuing Telemetry Transport (MQTT)* is a lightweight publish/subscribe protocol optimized for machine-to-machine (M2M) communication and IoT applications. Clients publish messages under specific topics to a broker, which then distributes them to the relevant subscribers. Its simple structure—comprising fixed and variable headers and a payload—allows efficient operation under constrained bandwidth conditions. In addition, its three Quality-of-Service (QoS) levels support flexible communication requirements. MQTT also supports TLS/SSL encryption for secure communication, making it highly suitable for distributed systems and wireless environments [38].

### 2.2. Related work

Research on integrating industrial systems with cloud and IoT platforms has proliferated, primarily leveraging MQTT, OPC UA, and Modbus, often with Node-RED serving as a PLC–cloud gateway. Studies by [42] and [43] demonstrated PLC-to-cloud monitoring and control. Gavlas et al. [42] reported that IBM Cloud achieved lower latency than Ubidots and MQTT, with sub-second responses, and recommended Node-RED optimization. Nguyen-Hoang and Vo-Tan [43] controlled VFD hardware through PLCs with ≈100 ms average latency for 36 bytes, supporting S7, Modbus, MQTT, and REST over IBM and AWS. Extending this, [32] integrated PROFIBUS, HART, Ethernet, and Modbus at the edge, used S7/Modbus locally and MQTT (Node-RED on IBM Watson) to the cloud, and measured 216 ms pub–rec latency, suitable for non-critical processes.

Comparative evaluations of industrial communication protocols (ICPs) show mixed trade-offs. [31] compared OPC UA, Modbus, and S7 Ethernet/IP for machine tools: Modbus showed lower latency, but software complexity varied; tests were local with limited variables to avoid PLC overload. [41] contrasted Modbus, IEC 60870−5, OPC UA, and MQTT using device capabilities/design/customer feedback, noting MQTT’s dynamic IP use and adoption, but no performance/latency measurements. [34,44] found MQTT faster than OPC UA for pure data exchange, with RTTs ranging from < 100 ms to < 1 s depending on payload size and QoS. They emphasized the need for data-type tooling and message sequencing. [45] demonstrated that OPC UA has minimal network impact but significant CPU/RAM load on low-spec edge devices (local testbed with no latency). [46] utilized OPC UA for CNC data collection/visualization within a stack (NC kernel, SoftPLC, OPC UA server, and multiple clients), thereby improving monitoring but without conducting latency analysis.

In distributed/IoT contexts, [47] combined IEEE 1451 and IEC 61499 in the IIRA, finding that MQTT was more bandwidth-efficient and faster than HTTP, while HTTP offered higher reliability for specific real-time tasks. [48] compared MQTT over WebSocket vs TCP/IP: WebSocket was slightly slower (267–289 ms) due to masking/unmasking. [33] measured WebSocket and MQTT across Italy–Brazil servers, finding similar mean RTTs (< 300 ms) across payloads and recommending that future studies include CPU/RAM and device metrics, as well as involve IoT devices as clients.

Digital Twin (DT) applications in education emphasize feasibility over communication metrics. [49] presented case studies using MQTT, OPC UA, OpenPLC, CODESYS, Modbus on Azure; [50] focused on DT with S7 in a local setup; [26] built a Factory I/O virtual plant integrated with Siemens S7 via TIA Portal, used Node-RED to transfer S7 data and created an HMI with SVG images, enabling remote DT operation during COVID-19.

Parallel developments target education. [51] built a low-cost remote PLC programming setup (scheduler, remote desktop, GUI, camera) but noted resetting lab hardware after sessions is challenging remotely. [30] proposed a Unity3D PLC teaching platform enabling real-time control over a local network. [52] simulated a process with a simulated PLC and Node-RED + Ubidots over S7, manually testing correct readings/graphs; Node-RED enabled control and visualization, while Ubidots provided visualization only.

Collectively, these studies confirm the feasibility of cloud/IoT integration and underscore the importance of protocol choice. However, significant gaps remain, including reliance on local networks, latencies exceeding real-time thresholds, unidirectional data flows, and limited multi-metric evaluation (e.g., latency distributions, scalability, and server resources). Educational DT work rarely quantifies low-latency bidirectional performance, synchronization accuracy, or resource efficiency in remote settings.

This study addresses these gaps with a rigorous, multi-metric evaluation of high-frequency, bidirectional, real-time communication between Siemens PLCs and cloud-hosted Digital Twin Prototypes (DTPs). We introduce and benchmark a WebSocket–S7 bridge (event-driven signaling) directly against MQTT, OPC UA, and Modbus, measuring round-trip latency, throughput, server resource usage, and tail performance (p95/p99), and validating in a multi-user educational scenario. These results provide practical guidance for robust, real-time DT deployments in industrial and academic contexts.

## 3. Experiment Set-up: Materials and methods

This section outlines the experimental setup and environment used to evaluate the protocols, the performance metrics, the data collection, and analysis process.

### 3.1. Experimental methodology

The experiment assessed the performance of multiple communication protocols within a Digital Twin Prototype (DTP) developed in Unity. The application, deployed on an Android device, used C# scripts built with protocol libraries for MQTT, WebSocket, OPC UA, and Modbus. These scripts established connections with a Node-RED server hosted on AWS, which interfaced with a local Node-RED instance connected to a Siemens S7-1500 PLC. When a start command was issued from the DTP, messages were transmitted to the AWS instance and relayed to the PLC for execution. PLC responses were returned through the same route, enabling closed-loop control of the automated box-sorting process in the virtual model.

A Siemens S7-1500 PLC and an AWS EC2 instance represented the industrial control and cloud components, respectively. The architecture is platform-agnostic: the WebSocket–S7 bridge and Node-RED integration can be adapted to other PLC brands (e.g., Allen-Bradley, Beckhoff) or cloud providers (e.g., Azure, Google Cloud) with minimal configuration changes.

Performance metrics—latency, throughput, and reliability—were collected through integrated time-stamping in the C# scripts and Node-RED functions. Additional traces were obtained using Siemens TIA Portal Long-Term Trace. JMeter was used for load emulation, Wireshark for packet inspection, and RStudio for statistical analysis and visualization. Resource utilization was monitored using the Unity Profiler on the Android device and AWS CloudWatch on the server side.

### 3.2. System design

**Fig 1:**
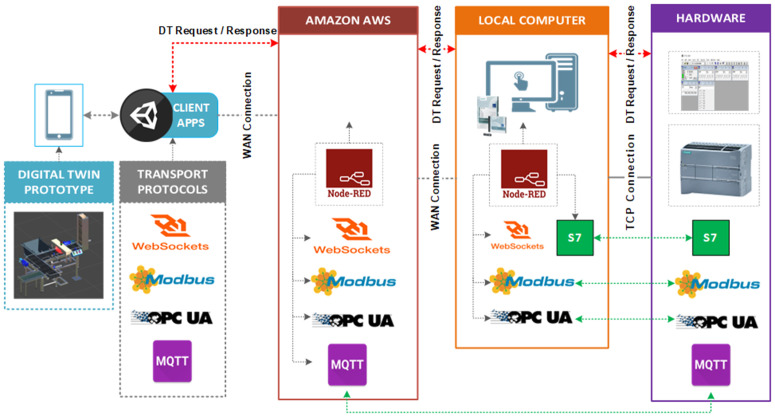
System framework.

Detailed hardware specifications for the experimental setup are listed in S1 Table. The system framework, shown in Fig 1, comprised three primary components:

- **Testbed**: Consisted of an Android mobile device, a computer with Unity installed, and a mobile application containing MonoBehaviour C# scripts developed to facilitate communication between the PLC and the 3D virtual model.
- **Node-RED**: Deployed on both an Amazon AWS EC2 instance and a local computer, serving as the middleware for message transfer between the DTP application and the Siemens PLC.
- **Siemens PLC**: A Siemens S7-1500 controller executing a ladder logic program to control the DTP model deployed on the Android device.

#### 3.2.1. Testbed.

The DTP application, developed using *Unity 3D 2022.3.17f1*, represents a virtual, automated box-sorting plant that simulates industrial operations, including piston activation, conveyor transport, and box inspection. The process begins when the start button is pressed, activating sensors and pistons that move boxes along a conveyor. A detection unit scans box dimensions and separates damaged boxes, which are removed by a pneumatic actuator. Intact boxes proceed to a second conveyor, and a final piston transfers them to the packaging stage.

**Fig 2:**
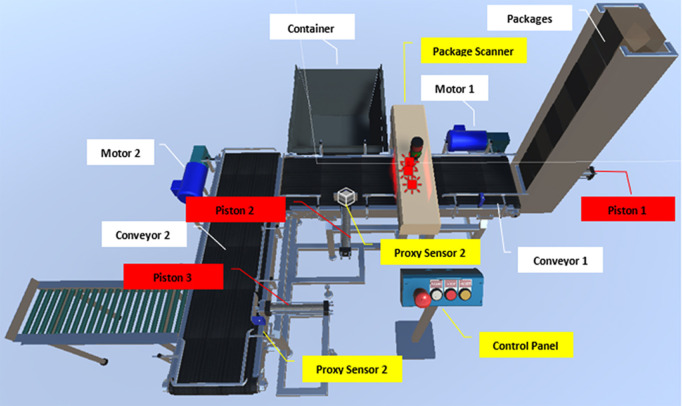
Testbed 3D model – automated box sorting plant.

The DTP–PLC communication relies on a combination of data transfer protocols, IoT cloud services, and industrial communication interfaces that enable bidirectional data exchange between the virtual environment and the physical controller. The 3D models of the automated plant (Fig 2) were created in SolidWorks.

Due to file-format compatibility issues, the models were refined in Blender to adjust hierarchy, coordinates, and scaling before being exported to FBX format to preserve textures and colors for Unity integration.

**Fig 3:**
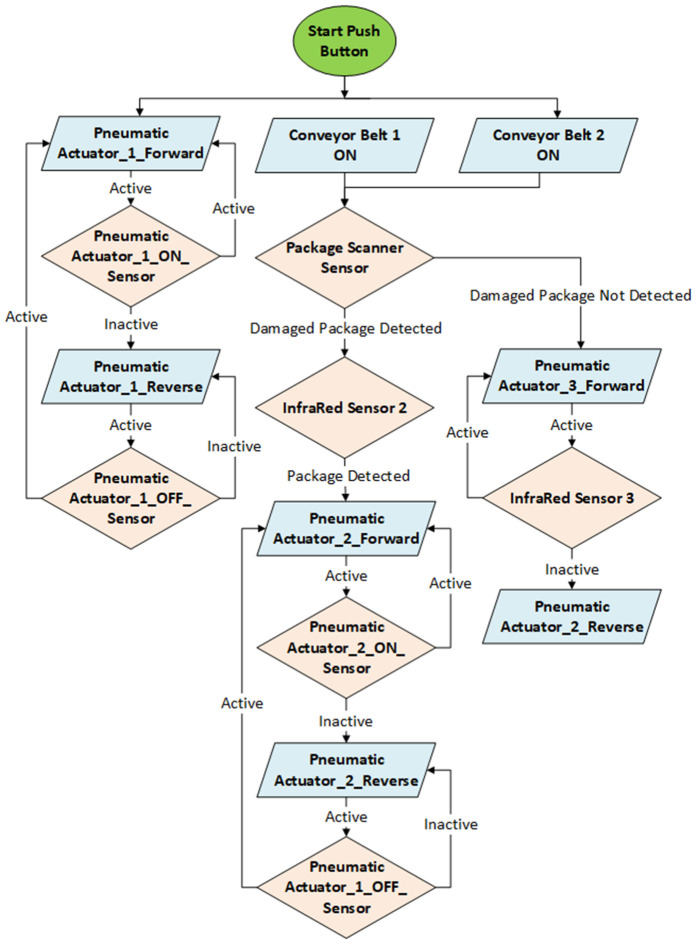
Testbed logic flowchart.

The complete process flow is illustrated in Fig 3. Each I/O element continuously communicates with the PLC using the configured protocol, as detailed in S2 Table. Data writing occurs in response to system events, such as sensor activation or button presses, triggering actions like conveyor movement or piston control. Timing measurements were implemented using Unity’s *System.Diagnostics.Stopwatch* class to record high-resolution latency data for each component, which was then exported to Excel for subsequent analysis.

All experiments were conducted across an intercity WAN connection. The Siemens S7-1500 PLC and AWS EC2/Node-RED server were located in Abu Dhabi (UAE), while the Unity-based DTP client operated in Al Ain, approximately 150 km away. This topology was kept constant across all protocol trials; no LAN shortcuts or direct connections were used.

To ensure that timing data reflected the full control-loop performance rather than isolated message latency, all measurements were recorded after completing an entire PLC execution cycle. Each valid run required the successful transmission of all command and feedback messages within the same cycle—any message loss or delay invalidated the result. Only fully synchronized and functionally complete cycles were included in the statistical analysis.

#### 3.2.2. Protocol libraries and optimizations.

**WebSocket Implementation.** The *WebSocketSharp* library, integrated within the Unity framework, was employed to establish real-time communication between the virtual model and the physical PLC system. Two WebSocket instances—*wsOutgoing* and *wsIncoming*—managed the transmission and reception of JSON-formatted data. Incoming messages dynamically updated the states of virtual actuators, ensuring that the digital twin remained synchronized with the live PLC-controlled system.

All WebSocket experiments used standard unencrypted WS connections over TCP (port 1880) to isolate protocol performance from encryption overhead. This deliberate exclusion of TLS ensured that latency results reflected pure transport characteristics rather than cryptographic delay. In production settings, equivalent WSS configurations can be activated in Node-RED with only marginal latency increases, which future industrial-scale tests will quantify.

**WebSocket Optimization for Real-Time Digital Twin Integration.** To meet the IEC 61588 real-time constraint (≤100ms round-trip time), several transport- and data-level optimizations were implemented in the Unity–PLC integration. These enhancements focused on reducing latency, improving stability under multi-user conditions, and ensuring deterministic synchronization between the digital and physical environments.

At the transport layer, the optimized *WebSocketSharp* library maintained persistent sessions, eliminating repeated handshake overhead. TCP parameters were tuned by enabling *TCP_NODELAY* (disabling Nagle’s algorithm) and turning off per-message deflate compression, which previously introduced jitter for small telemetry frames. Session continuity across WAN conditions was sustained through periodic *PING/PONG* keep-alives, exponential backoff for reconnection, and jitter-based retry scheduling. On the server side, event-loop backpressure and frame coalescing mechanisms stabilized fan-out during multi-user sessions, preventing lagging clients from generating queue growth or instability under classroom-scale loads.

At the data layer, serialization overhead was minimized by using a structured message envelope that contained sequence numbers and timestamps, supporting both ordered delivery and deduplication. Compact JSON payloads were retained for interoperability with Unity scripts and Node-RED, while preserving a binary-ready schema for future extensions. Asynchronous, non-blocking I/O replaced synchronous calls to avoid frame stalls, and buffer pooling mitigated garbage collection overhead on mobile and AR devices. Variable names were abbreviated, and multiple I/O variables were batched per frame to reduce payload size. Whenever possible, update timing was synchronized with the PLC scan cycle, aligning message grouping with deterministic control logic. Although binary frames offered slightly lower serialization latency, JSON was preserved for transparency and ease of debugging—consistent with industrial integration practices that favor human-readable telemetry across heterogeneous platforms.

These optimizations produced measurable performance gains. Compared with the baseline configuration, the tuned WebSocket channel exhibited markedly reduced jitter, improved p95/p99 stability, and near-zero session drops under multi-user conditions. Disabling Nagle’s algorithm via *TCP_NODELAY* and batching multiple I/O variables per frame yielded the most considerable latency improvements. These measures lowered the average round-trip time by approximately 25–35%. Persistent session management and transport-level tuning further enhanced stability, cutting jitter by more than half under WAN load. Collectively, these measures enabled deterministic and responsive synchronization between the Unity-based digital twin and the Siemens PLC across both local and cloud-mediated environments.

**OPC UA Implementation**. The *Opc.Ua.Client* library was used to manage OPC UA connections, initializing a client session and employing Node IDs for efficient data transmission. Data handling involved direct server interactions through methods such as *Session.Read* and *Session.Write*. Data retrieval was implemented via a coroutine executed at fixed intervals (50 ms) to ensure timely updates. However, reducing polling intervals further was found to impose excessive network and computational loads, outweighing potential latency gains and affecting system stability. Additional tests using OPC UA subscriptions instead of polling were also conducted, but they exhibited higher latency. This reaffirmed the chosen polling approach as the most effective for maintaining low delays in real-time applications.

**Modbus Implementation.** The *Modbus_Client* class in Unity, implemented using the *EasyModbus* library, handled real-time communication with the Modbus server controlling the PLC system. The client connected to a predefined IP address and port, employing *ReadHoldingRegisters* for data retrieval and *WriteSingleRegister* for command transmission. Data reading was performed through a coroutine executing every 50 ms, balancing update frequency with network efficiency. Sixteen-bit holding registers were used to encode both boolean and numerical values, with boolean states compacted via bitwise operations—maximizing register utilization for precise control and monitoring.

**MQTT Implementation.** The *MqttClient_* class in Unity utilized the *uPLibrary.Networking.M2Mqtt* library to manage MQTT communication. The script initialized an MQTT client, defined subscription and publication topics, and encoded boolean states into byte arrays for efficient payload handling. Data publishing, driven by state changes such as sensor activations or button presses, was executed using the *Publish* method at QoS 0 to minimize latency. Incoming MQTT messages were received via an event handler that decoded the payloads and updated system variables in real time.

**Failure Handling and Robustness Considerations.** While this study primarily focused on performance benchmarking under normal operating conditions, several mechanisms were implemented to preserve real-time stability during transient network disturbances. The WebSocket configuration incorporated automatic reconnection logic with exponential backoff intervals (200 ms, 400 ms, and 800 ms). It also used periodic *PING/PONG* keep-alive frames every 30 s to detect half-open connections, along with deterministic session recovery using sequence-numbered payload envelopes. These mechanisms ensured that temporary disruptions triggered bounded recovery without requiring user intervention. For MQTT, a Quality of Service (QoS) level 0 was selected to prioritize latency over delivery guarantees, consistent with real-time control applications where outdated data is less valuable than timely updates. Although the system demonstrated robust reconnection during incidental WAN fluctuations, comprehensive failure-mode testing—including extended network partitions, packet loss, and graceful degradation strategies—remains beyond the scope of this performance-focused study and is identified as future work toward production-grade robustness.

**Protocol Validation.** All protocol scripts were validated in a local Node-RED test environment independent of AWS, confirming reliable communication and sub-100 ms latencies across all protocols. This validation verified that each implementation meets the real-time performance requirements necessary for precise, low-latency operation in automated control systems.

#### 3.2.3. PLC configuration.

The experimental setup utilized a Siemens S7-1500 PLC, which was configured and programmed using the TIA Portal environment. The PLC was connected to a computer via Ethernet, assigned a static internal IP address, and linked to the internet through a high-speed network connection.

The ladder logic program (S6 Fig) was downloaded to each PLC instance to enable protocol-specific communication with the DTP. While the PLCs shared common program blocks and functions, their communication libraries were customized according to the protocol under evaluation. This ensured compatibility and efficient data exchange between the PLC and the digital twin environment.

For MQTT communication, Siemens’ external *LMQTT_Client v4* library was used in conjunction with dedicated Function Calls (FCs) to encapsulate and decapsulate incoming and outgoing traffic. Incoming MQTT messages were unpacked into input byte arrays via the *SCATTER* function and mapped to internal PLC variables. Conversely, PLC states were packed into output tag arrays using the *GATHER* function, which assembled MQTT message payloads for transmission.

For OPC UA and Modbus communication, the Siemens S7-1500 operated as an integrated OPC server. In the Modbus configuration, the *Modbus Server Communication v5.3* library was employed alongside dedicated FCs to encapsulate data into 16-bit word formats and decapsulate incoming traffic into PLC variables. This modular structure ensured consistent message handling across all protocol types, enabling standardized testing and comparative performance evaluation.

#### 3.2.4. Node-RED platform configuration.

The experimental architecture leveraged Node-RED and AWS EC2 to coordinate communication between the DTP application and the PLC. Node-RED is a flow-based programming tool optimized for IoT integration. It enables the rapid customization and deployment of protocol flows through a visual interface, thereby reducing development time and improving interoperability. It natively supports MQTT, HTTP, WebSocket, Modbus, and OPC UA, providing real-time data exchange and remote monitoring through its extensive component library.

The cloud infrastructure was built on an AWS EC2 Linux instance hosting Node-RED, which served as the central coordination hub. The use of a public IP address eliminated the need for static IP addresses, VPNs, port forwarding, or complex firewall configurations. This approach simplified access and enhanced scalability. The EC2 instance was hosted in a local AWS region to minimize latency and maximize throughput, ensuring consistent and reliable real-time performance.

In this setup, each PLC communicated with a locally installed Node-RED instance, which in turn relayed messages to the cloud-hosted Node-RED on AWS EC2. This configuration created a robust communication bridge between the physical controllers and the DTP running on Android, with protocol-specific flows ensuring data integrity and bidirectional responsiveness. MQTT messages were routed directly between the endpoints, benefiting from MQTT’s lightweight structure and avoiding the need for complex static IP or port-forwarding configurations typically required by other protocols.

**Fig 4:**
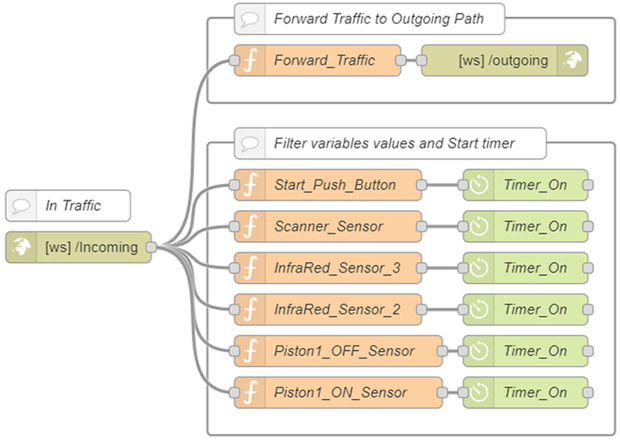
AWS Node-RED flow with WebSocket-Out node.

**Fig 5:**
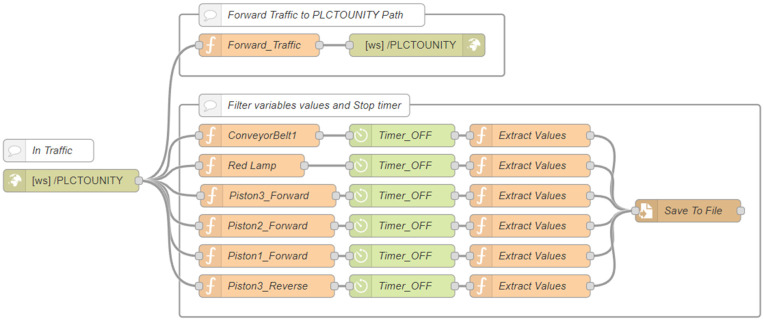
AWS Node-RED flow with WebSocket-In node.

**Node-RED with WebSocket.** The AWS Node-RED instance implemented a bidirectional communication pathway (Figs 4–5). A *WebSocket In* node, configured on the “*Incoming*” path, received messages from the DTP client. These were relayed to the local Node-RED instance via a corresponding *WebSocket Out* node configured on the “*Outgoing*” path.

A specialized subflow named *flow-timer* included a timer function node and a custom function that triggered timing events based on message activity. This mechanism measured response times from the PLC and stored them for analysis, offering precise latency data and enabling real-time performance diagnostics. A dedicated *WebSocket In* node with the path “*PLCTOUNITY*” forwarded PLC messages to the DTP client. Complementary function nodes stopped timers and logged time-stamped results when corresponding PLC responses were received, ensuring synchronization between the physical and virtual systems.

**Fig 6:**
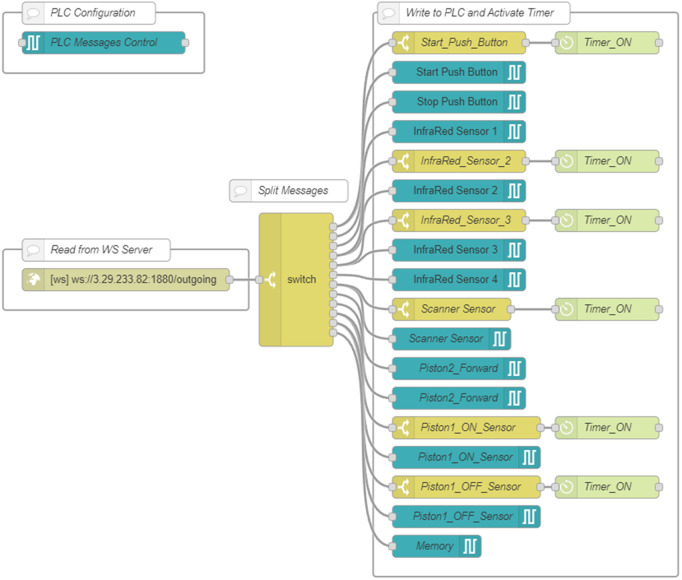
Local Node-RED flow with WebSocket-In node and S7 node.

**Fig 7:**
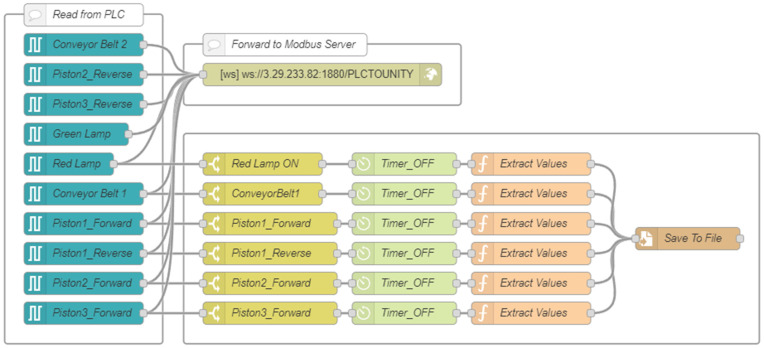
Local Node-RED flow with WebSocket-Out node and S7 node.

On the local Node-RED instance, a custom flow combining WebSocket and S7 protocols managed communication with the PLC (Figs 6–7). A *WebSocket In* node, configured to connect to the AWS WebSocket server, captured incoming messages containing structured variable names and payloads. These were routed via switch and function nodes to the appropriate *S7 Control* nodes, where each variable was processed according to its address, ensuring accurate data alignment between systems.

A direct PLC link was maintained through *S7 Out* nodes, while messages from the PLC—captured by *S7 In* nodes—were transmitted back to AWS in real time through a *WebSocket Out* node using the “*PLCTOUNITY*” path. This configuration minimized network load by forwarding only event-driven data updates, maintaining tight synchronization across the distributed environment.

**Node-RED with OPC UA.** The OPC UA configuration on AWS was designed to leverage the platform’s capabilities as an OPC UA server (S1 Fig). Within Node-RED, the OPC UA server node was configured to use the public IP address of the EC2 instance hosting the AWS environment, allowing remote access and interoperability with external clients.

To initiate the data exchange process, variables were injected into the system at startup using *inject* nodes. Each variable was uniquely identified by a NodeId (i) and a Namespace index (ns), which were essential for distinguishing and referencing nodes in the address space. By defining these identifiers, client applications could seamlessly subscribe to designated variables and receive timely value updates, enabling real-time monitoring and control.

Additional OPC UA client nodes were strategically deployed within the AWS configuration to subscribe to these variables, enabling the triggering and cessation of timer functions used to measure the round-trip communication time between the AWS OPC UA server and the PLC.

On the local Node-RED instance, a complementary OPC UA flow orchestrated communication with the AWS server. Input variable values were retrieved through “OPCUA Item” and “OPCUA Client” nodes, which were configured with a rapid 10 ms subscription interval to ensure real-time data acquisition. Once the variable status was updated, the values were transmitted to the PLC, which functioned exclusively as an OPC UA server. This arrangement provided flexibility, allowing the PLC to both publish and consume data from other OPC UA clients.

The “Write OPCUA Client” node handled data transmission to the PLC, ensuring seamless updates of PLC variables (S2 Fig). Conversely, outgoing messages originating from the PLC were captured by a “Subscribe OPCUA Client” node and relayed to a “Write” node on the AWS server side, maintaining bidirectional communication and consistent variable synchronization across cloud and local layers (S3 Fig).

**Node-RED with Modbus.** In the Modbus configuration, the AWS EC2 instance hosted a Modbus server configured with its public IP address as the endpoint (S4 Fig). Two *Modbus Read* nodes accessed holding registers at an 80 ms interval to log start and stop times for latency measurement. Address 0 was assigned for receiving messages from the DTP, while Address 1 was used for transmitting data to the PLC. This clear separation of read and write addresses ensured precise, collision-free data flow and enhanced system reliability during bidirectional exchanges between the DTP and PLC.

On the local Node-RED instance, *Modbus Read* nodes retrieved data from holding registers on the AWS Modbus server at the same 80 ms interval. Attempts to reduce this interval resulted in noticeable transmission delays, confirming 80 ms as the optimal balance between responsiveness and network stability. Detected variable changes were transmitted to the PLC using *Preset Multiple Register* nodes, creating a direct link for real-time bidirectional communication.

In a parallel process, another *Modbus Read* node continuously polled the PLC at 80 ms intervals, forwarding updated values to the AWS server via *Modbus Write* nodes (S5 Fig). This closed-loop configuration maintained consistent state synchronization across the distributed environment while minimizing network load and ensuring deterministic timing.

### 3.3. Evaluation tools and methods

#### 3.3.1. Profiling and performance measurement.

**Unity Profiler analysis.** *Unity Memory Profiler v1.1.0* and *Profile Analyzer v1.2.2* were used to evaluate the DTP application’s performance on Android devices. Data were collected via USB from the *Unity 2022.3.17f1* environment, with “Development Build” and “Autoconnect Profiler” enabled to ensure communication with the mobile device. Real-time monitoring of CPU and RAM utilization enabled the identification of performance bottlenecks and overall application efficiency.

**Round-Trip Time measurement.** Message latency between the Unity DTP application and the remote PLC, transmitted through AWS Node-RED, was measured using a *Stopwatch()* function in Unity to record the interval from input activation to PLC response, with the results stored in CSV format. Concurrently, Siemens *TIA Portal V18 Long-Term Trace* logged controller-side timestamps, while Node-RED recorded variable write and response times. This multi-point measurement provided a detailed decomposition of each communication stage and guided protocol optimization for real-time performance.

#### 3.3.2. Network and server monitoring.

**Wireshark network analysis.** *Wireshark v4.2.4* captured and analyzed network traffic during protocol testing. Packet captures targeted specific server IPs and ports for each protocol, allowing for the precise isolation of communication events. Wireshark’s inspection tools revealed latency distributions, retransmissions, and anomalies across protocol implementations.

**AWS resource monitoring.** AWS EC2 instances (running Amazon Linux 2023) hosted Node-RED as the servers for Modbus, MQTT, WebSocket, and OPC UA protocols. The *AWS CloudWatch Agent* monitored CPU usage, memory consumption, and network bandwidth, augmented with custom metrics for memory utilization beyond default AWS monitoring. These data characterized server-side efficiency, throughput, and stability under variable load conditions.

#### 3.3.3. Load and scalability testing.

Protocol scalability and stability were evaluated using *Apache JMeter v5.6.3*, simulating multi-user scenarios to assess throughput, latency, and error rates. Load tests were executed with 1, 5, 10, 20, 40, 60, and 80 virtual users to represent different concurrency levels. JMeter’s listeners and timers captured detailed metrics on protocol responsiveness and server resource impact.

- *WebSocket and MQTT:* Dedicated thread groups used WebSocket and MQTT samplers aligned with DTP process sequences. MQTT samplers simulated publish/subscribe interactions via custom scripts for payload encoding and message sequencing.
- *OPC UA and Modbus:* Because JMeter lacks native support for these protocols, custom samplers were developed in Java using the *Eclipse Milo* library for OPC UA and *j2mod* for Modbus. These samplers managed connection setup, read/write operations, and response validation. Full implementation details and source code are available in [53,54].

Error rates were validated using *Response Assertion* elements in JMeter, comparing returned variable states with expected PLC outputs. Flow-control elements and synchronized timers replicated DTP operating dynamics, ensuring consistency between simulated and real PLC interactions.

#### 3.3.4. Data collection and reproducibility.

All raw measurement data were systematically archived for reproducibility. Unity C# scripts logged round-trip times with microsecond precision into timestamped CSV files annotated by protocol and test condition. JMeter results were exported in JTL (CSV) format, Wireshark captures in PCAP format with protocol-specific filters, and AWS CloudWatch metrics as JSON time series. All datasets, analysis scripts, and configuration files are stored in a structured repository, with the custom JMeter samplers publicly available in [53,54] to support independent replication of the load-testing framework.

## 4. Results and evaluation

The system’s performance and functionality were evaluated through a multi-faceted approach using Wireshark, JMeter, AWS CloudWatch, and the Unity Profiler to assess communication latency, stability, and resource utilization.

### 4.1. Round-trip latency analysis

Analysis of the communication protocol round-trip data (Table 1) revealed clear trends in latency across different days and server conditions. Visualizations illustrate the distribution and summary statistics of elapsed times per protocol. Box plots depict the dispersion of elapsed times for each protocol, with a dashed red line representing the overall mean. Line plots summarize the mean, median, minimum, and maximum elapsed-time values.

**Table 1:** Summary statistics of elapsed time per variable condition.

Protocol	Variables	Metrics (ms)									
		Average	Median	Min	Max	Count	Std_Dev	Range	Q1	Q3	IQR
MQTT	Conveyor Belt	136	135	52	242	100	39	190	106	162	56
	Piston1 Backward	130	124	38	256	900	38	218	101	159	58
	Piston2 Forward	136	131	51	246	400	38	195	105	162	57
	Piston3 Forward	136	137	56	252	400	37	196	108	161	53
	Red Lamp	135	136	60	248	400	38	188	106	160	54
	**Overall Average**	**133**	**130**	**38**	**256**	**2200**	**38**	**218**	**103**	**160**	**57**
Modbus	Conveyor Belt	195	197	104	284	100	36	180	168	219	51
	Piston1 Backward	193	191	89	336	900	41	247	164	219	55
	Piston2 Forward	194	194	100	323	400	43	223	164	217	53
	Piston3 Forward	197	199	94	306	400	42	212	165	224	59
	Red Lamp	190	190	19	301	400	45	282	162	219	57
	**Overall Average**	**194**	**194**	**19**	**336**	**2200**	**42**	**317**	**164**	**219**	**55**
OPC UA	Conveyor Belt	175	171	63	286	100	44	223	148	186	39
	Piston1 Backward	170	168	63	290	900	36	227	148	181	33
	Piston2 Forward	164	156	76	290	400	34	214	141	178	37
	Piston3 Forward	149	149	28	280	400	33	252	130	169	39
	Red Lamp	151	150	48	256	400	30	208	131	171	40
	**Overall Average**	**162**	**159**	**28**	**290**	**2200**	**35**	**262**	**139**	**178**	**39**
WebSocket	Conveyor Belt	83	86	30	119	100	17	89	68	93	26
	Piston1 Backward	87	88	45	136	900	16	91	75	98	23
	Piston2 Forward	88	90	51	119	400	15	68	78	99	21
	Piston3 Forward	85	87	48	116	400	15	68	75	97	22
	Red Lamp	90	92	51	116	400	15	65	79	101	22
	**Overall Average**	**87**	**88**	**30**	**136**	**2200**	**16**	**106**	**76**	**99**	**23**

To establish a reliable network baseline, ICMP latency between the Al Ain client and the Abu Dhabi EC2 server was measured regularly—averaging 200 samples per day—and remained stable between 18 ms and 21 ms.

**Fig 8:**
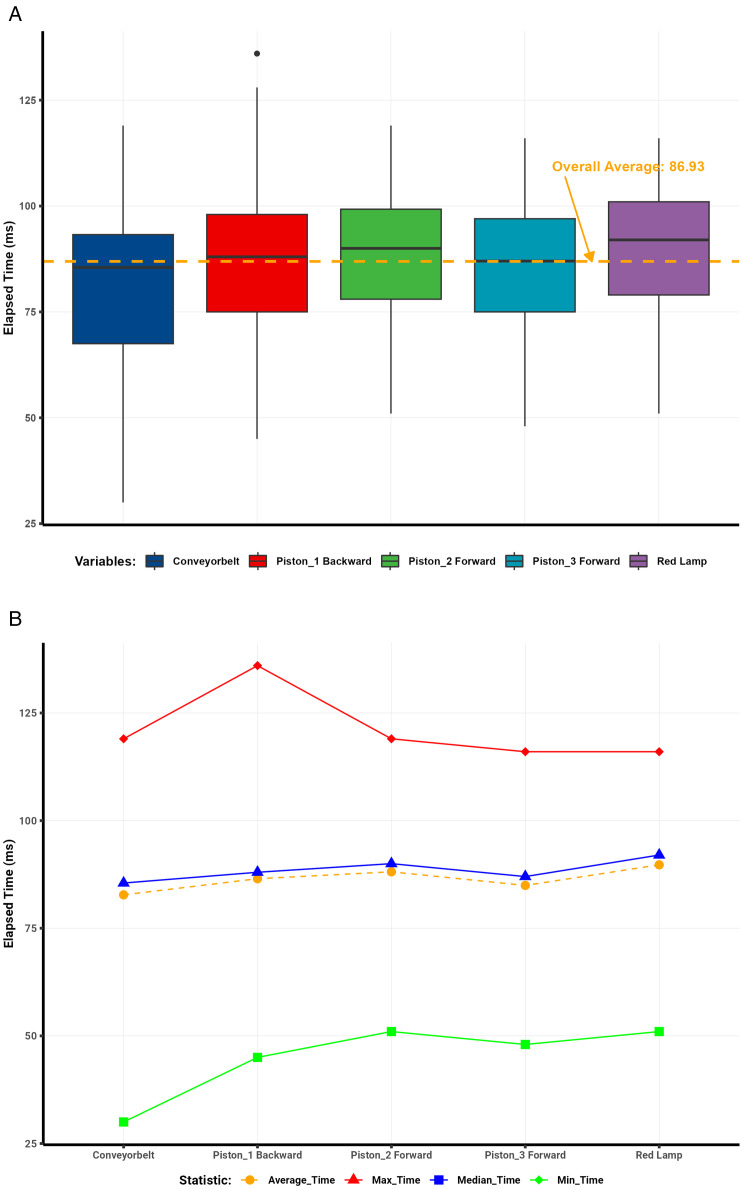
WebSocket round-trip latency analysis. (a) WebSocket round-trip latency distribution. (b) WebSocket summary statistics of elapsed time.

The WebSocket protocol demonstrated the most consistent performance, achieving an average round-trip time of 87 ms and a median of 88 ms across 2,200 interactions (Figs 8a–8b). The standard deviation of 16 ms indicated minimal variability, with only isolated outliers caused by brief network congestion or server delays.

**Fig 9:**
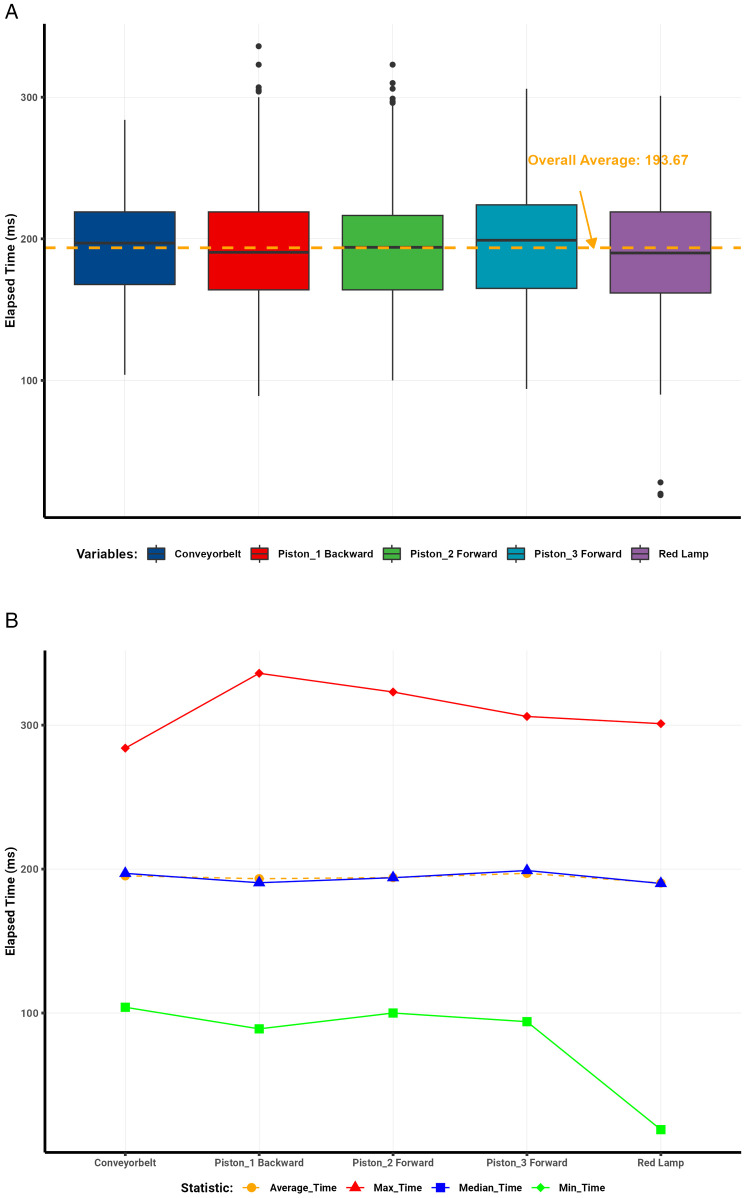
Modbus round-trip latency analysis. (a) Modbus round-trip latency distribution. (b) Modbus summary statistics of elapsed time.

In contrast, Modbus showed greater variability (Figs 9a–9b), with mean and median round-trip times of 194 ms (SD = 42 ms). Numerous outliers reflected instability from its serial communication design and intermittent network delays.

**Fig 10:**
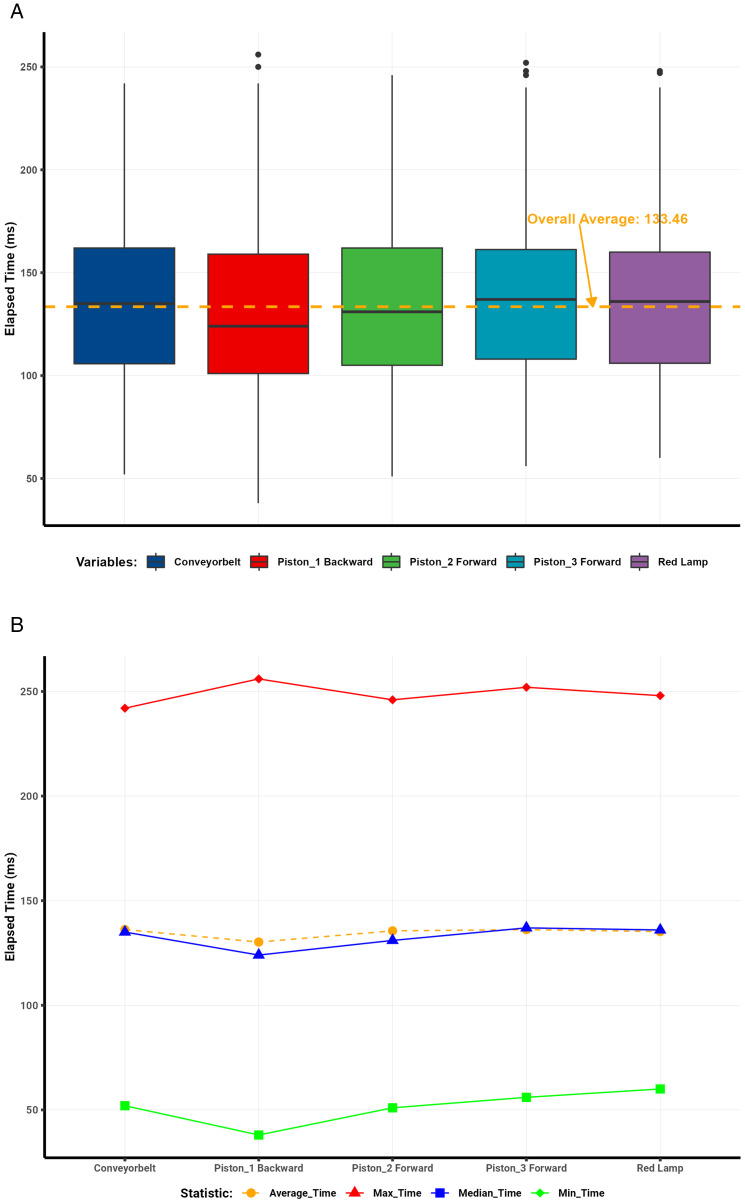
MQTT round-trip latency analysis. (a) MQTT round-trip latency distribution. (b) MQTT summary statistics of elapsed time.

MQTT achieved moderate performance (Figs 10a–10b), with an average round-trip time of 133 ms, a median of 130 ms, and a standard deviation of 38 ms. Latency ranged between 38 ms and 256 ms. The outliers observed were primarily attributed to broker processing delays and transient network conditions.

**Fig 11:**
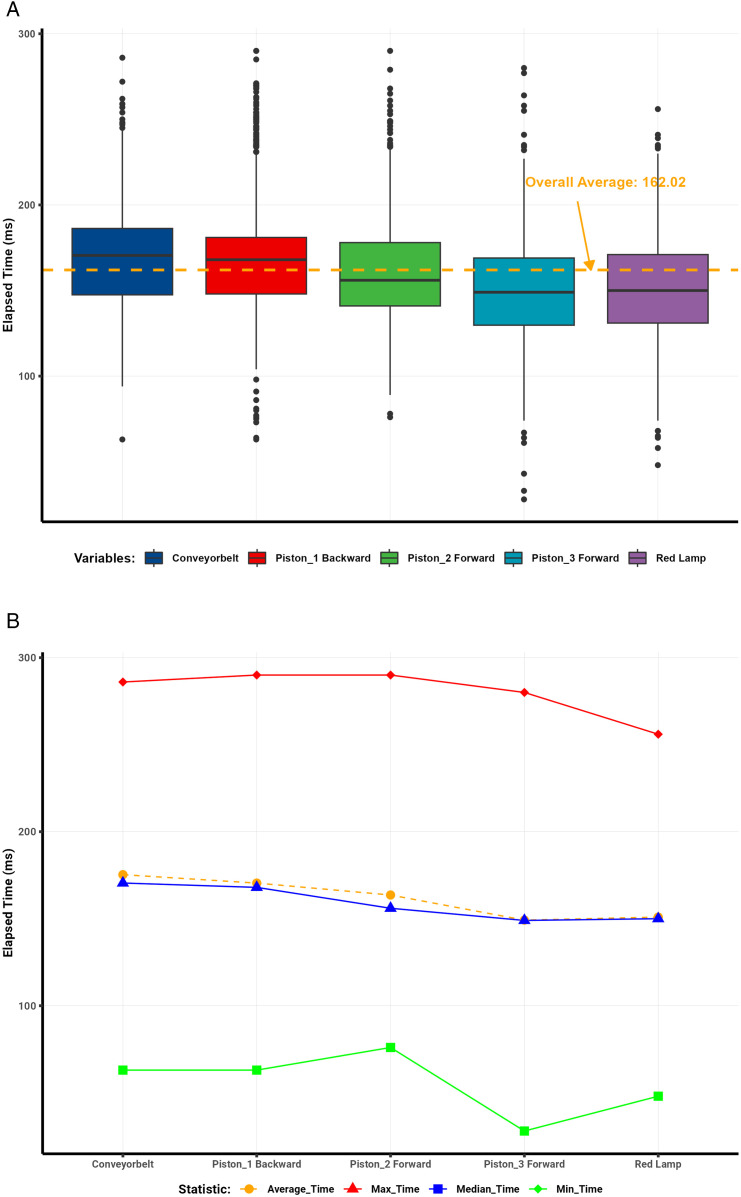
OPC UA round-trip latency analysis. (a) OPC UA round-trip latency distribution. (b) OPC UA summary statistics of elapsed time.

Similarly, OPC UA showed intermediate results (Figs 11a–11b), with an average latency of 162 ms, a median of 159 ms, and a standard deviation of 35 ms. Elapsed times ranged from 28 ms to 290 ms, with occasional outliers likely caused by the protocol’s complex data modeling and encoding overhead.

Overall, the comparative results confirm that the WebSocket–S7 bridge outperformed all other protocols in latency and stability, achieving the lowest average round-trip times and minimal jitter. This consistency makes it particularly suitable for high-frequency, real-time industrial applications requiring deterministic communication. Modbus, although functional, exhibited the most significant delay variance, rendering it less ideal for high-speed control. MQTT and OPC UA provided acceptable but less consistent performance, positioning WebSocket as the preferred protocol for time-critical digital twin synchronization.

### 4.2. Complete time analysis

The complete cycle time—from pressing the start button to the successful sorting of all boxes—was recorded using the *TIA Portal Long-Term Trace* tool. This metric reflects the total system responsiveness and was influenced by the conveyor and piston speeds, which were iteratively adjusted to determine the highest operational speed achievable without sorting errors.

**Fig 12:**
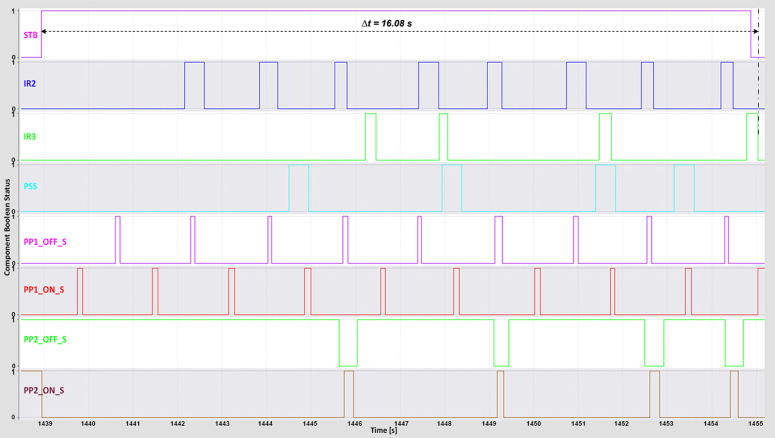
WebSocket complete time TIA Portal long-term trace.

**Fig 13:**
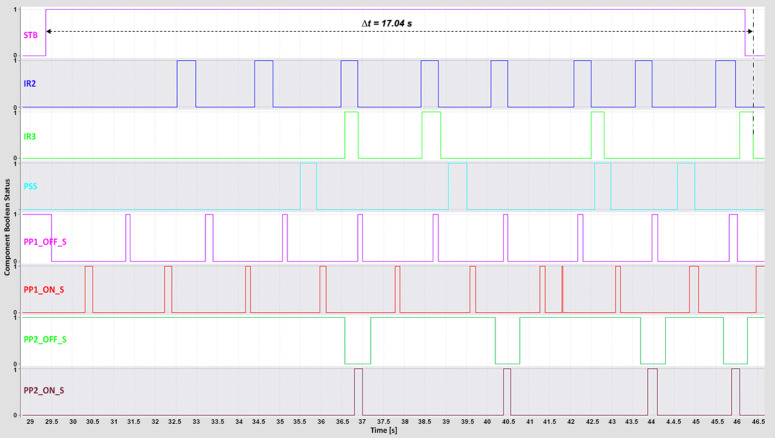
MQTT complete time TIA Portal long-term trace.

For the WebSocket and MQTT protocols, the minimum complete cycle times were 16 s and 17 s, respectively (Figs 12–13).

These results highlight both protocols’ ability to maintain low-latency, stable communication under optimal conditions. The DTP’s 3D model operated smoothly even at higher speeds, indicating that reduced latency directly contributed to precise motion control and synchronized feedback. The WebSocket protocol, in particular, maintained consistent performance during rapid request–response exchanges, thereby preserving system stability and responsiveness at high throughput levels.

**Fig 14:**
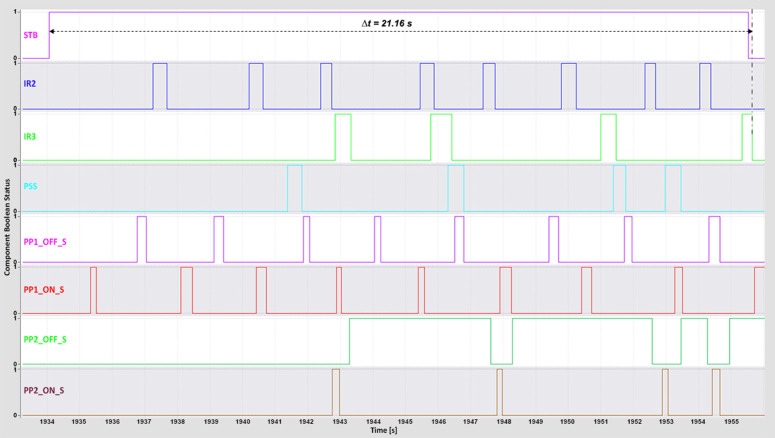
OPC UA complete time TIA Portal long-term trace.

**Fig 15:**
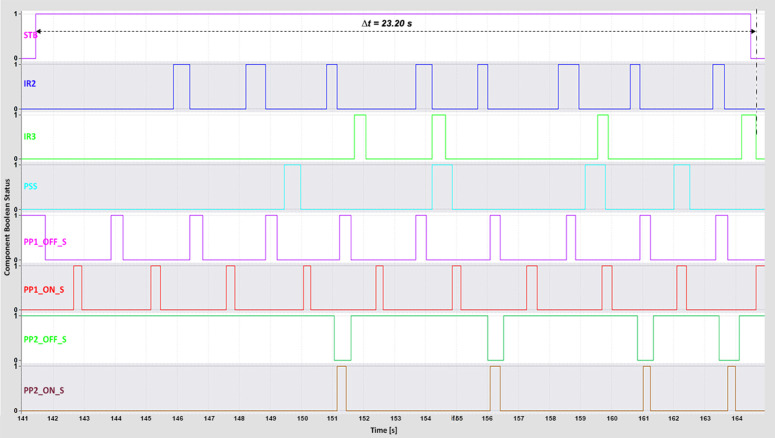
Modbus complete time TIA Portal long-term trace.

The OPC UA protocol achieved a minimum complete cycle time of 21 s (Fig 14), performing reliably but exhibiting minor irregularities at higher conveyor speeds. Modbus recorded the longest minimum complete cycle time of 23 s (Fig 15), with noticeable performance degradation under increased speed conditions. The delays in PLC response led to less fluid machine behavior and intermittent pauses during sorting. These effects highlight Modbus’s limitations in handling rapid, high-frequency communication compared with WebSocket and MQTT, which maintain smoother and more deterministic operation.

### 4.3. End device CPU frame time and memory usage analysis

CPU frame times were measured using the *Unity Profiler* on an Android device to evaluate the DTP application’s runtime performance under WebSocket, MQTT, OPC UA, and Modbus communication protocols. These metrics are crucial for assessing the simulation’s responsiveness and stability during real-time industrial automation.

The WebSocket protocol achieved the best performance, with a maximum frame time of 202 ms, a mean of 46 ms and a median of 46 ms, indicating smooth rendering and efficient task handling that maintained a highly responsive virtual environment. MQTT followed closely, with a maximum frame time of 2,250 ms, a mean of 48 ms and a median of 44 ms. Despite occasional spikes, its overall frame performance remained consistent and reliable.

OPC UA exhibited moderate efficiency, with a maximum frame time of 1,563 ms, a mean of 81 ms and a median of 72 ms, reflecting occasional rendering delays that slightly reduced visual smoothness. Modbus recorded the highest CPU frame times, with a maximum of 2,405 ms, a mean of 117 ms and a median of 100 ms. This indicates heavier communication overhead and reduced responsiveness. The elevated and more variable frame times observed for Modbus align with the less fluid motion of the 3D system, where delayed communication caused perceptible interruptions in component behavior.

Notably, higher CPU frame times were recorded during scene initialization and restart phases, corresponding to the establishment of PLC connections and variable initialization. These temporary spikes reflect the increased computational load associated with connection setup rather than sustained runtime inefficiency.

**Fig 16:**
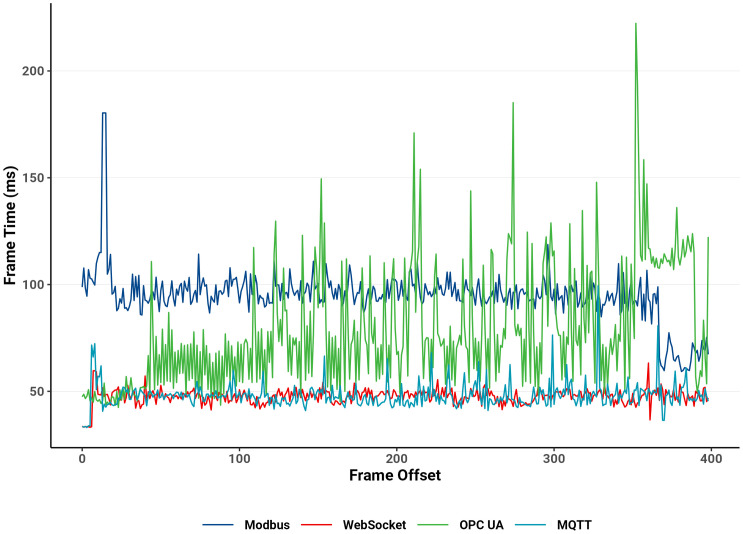
CPU usage analysis captured in Unity Profiler.

Overall, the pronounced differences in frame-time behavior—particularly in maximum latency—highlight the superior runtime efficiency of WebSocket and MQTT compared with OPC UA and Modbus. Their lower frame times supported smoother 3D motion, faster feedback, and greater simulation stability. These findings reinforce WebSocket’s suitability for real-time digital twin communication, ensuring seamless data exchange and reliable system responsiveness. Table 2 and Fig 16 summarize these frame-time results, excluding extreme outliers for clarity of comparison.

**Table 2:** Summary of CPU frame time.

Term	Frame Time (ms)			
	Websocket	Modbus	OPC	MQTT
Max	202	2405	1563	2250
Upper Quartile	49	104	88	48
Median	46	100	72	44
Mean	46	117	81	48
Lower Quartile	44	96	55	33
Min	19	83	39	23

### 4.4. Bandwidth usage analysis

Network traffic data for the WebSocket, MQTT, Modbus, and OPC UA protocols were captured and analyzed using *Wireshark*. The goal was to assess and compare the efficiency of each protocol in terms of bandwidth usage, throughput, and transmission patterns on the end device. Captured data were evaluated over multiple time intervals to identify trends, anomalies, and protocol-specific behaviors relevant to network optimization and protocol selection.

**Fig 17:**
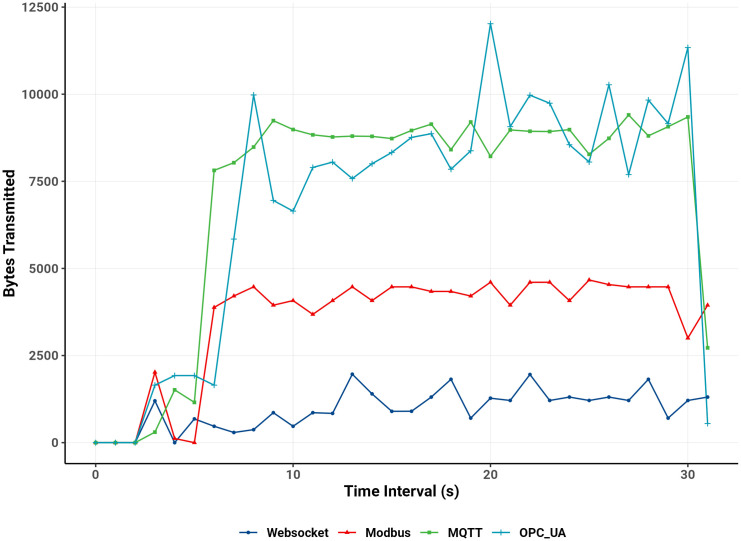
Bandwidth usage over time.

From these traces, the average, median, minimum, maximum, and standard deviation of transmitted bytes were calculated, and throughput was computed using Eq. 1. Table 3 summarizes these statistics, while Fig 17 illustrates bandwidth variation over time for each protocol.


$$\text{Throughput (Bytes per second)}=\frac{\text{Total Bytes Transmitted}}{\text{Total Time}}$$ (1)

**Table 3:** Network traffic data captured by Wireshark.

Protocol	Throughput (bytes/s)	Total		Size (byte)			
		Packets	Size [KB]	Mean	Min	Max	SD
WebSocket	993	195	30.8	962	292	1964	455
Modbus	3621	1717	112.3	3508	119	4668	939
OPC UA	6985	1302	216.5	6766	550	12022	3051
MQTT	7276	2265	225.6	7049	302	9405	2633

The WebSocket protocol exhibited the lowest overall bandwidth consumption, with an average throughput of approximately 993 bytes/s and a low standard deviation of 455 bytes. Across 195 packets totaling 31 KB, WebSocket maintained steady, lightweight transmissions by sending individual Boolean values encapsulated in JSON objects. This approach resulted in consistent, low-overhead communication. Efficiency could be further enhanced by batching multiple variables per JSON frame. This would reduce the packet frequency.

Modbus, which packs multiple Boolean values into a 16-bit word before transmission, achieved moderate throughput (3,621 bytes/s) and average packet size (3,508 bytes), with a total data volume of 112 KB across 1,717 packets. Its reduced message frequency and low overhead yielded stable and predictable transmission behavior, reflected in a moderate standard deviation (939 bytes). Modbus, therefore, balances throughput and stability effectively for applications with moderate data exchange requirements.

OPC UA achieved a similarly high throughput (6,985 bytes/s) with an average packet size of 6,766 bytes, transmitting 217 KB through 1,302 packets. Yet, it displayed significant variability, with a standard deviation of approximately 3,051 bytes. This fluctuation stems from OPC UA’s method of writing Boolean values individually to server nodes, which produces irregular intervals and greater sensitivity to network latency. Although capable of handling large data volumes, this complexity can lead to congestion if not properly optimized.

**Fig 18:**
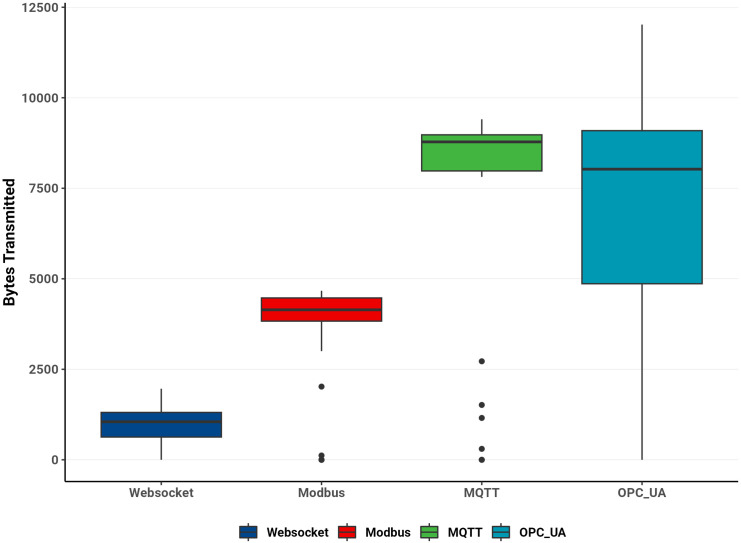
Distribution of bytes transmitted by protocols.

The box plot in Fig 18 visualizes the distribution of transmitted bytes, highlighting protocol differences.

WebSocket and Modbus showed narrower interquartile ranges and fewer outliers, confirming stable transmission. MQTT exhibited moderate variability, while OPC UA displayed a broader spread and more outliers, indicating greater transmission fluctuation.

Overall, these findings underscore distinct communication behaviors among the protocols. WebSocket’s frequent small transmissions require the least bandwidth and are well-suited to constrained networks or scenarios with frequent updates. Modbus and MQTT efficiently batch multiple values, achieving higher throughput at a moderate bandwidth cost. In contrast, OPC UA, while powerful for complex data models, demands careful configuration to maintain stable performance under varying network conditions.

### 4.5. Load analysis of performance metrics

**Fig 19:**
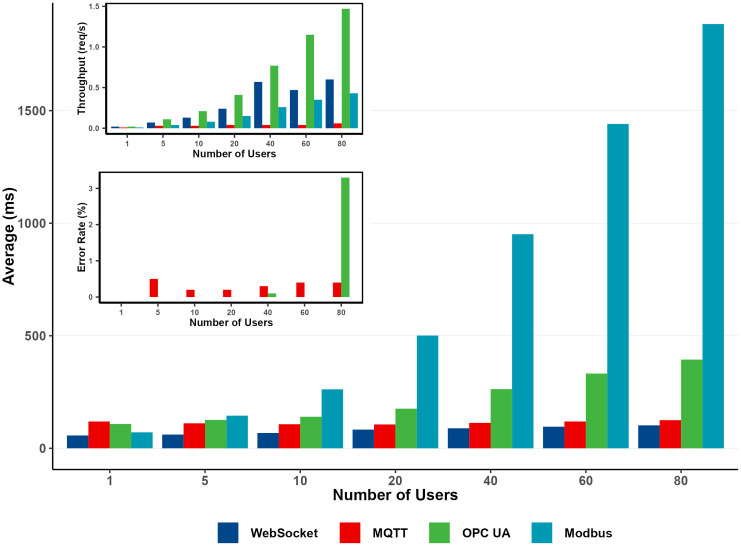
Average response time by user load.

This section presents a comparative evaluation of performance metrics obtained from JMeter tests using MQTT, OPC UA, Modbus, and WebSocket protocols. The analyzed metrics include average, minimum, and maximum response times, standard deviation, error percentage, and throughput across various user load levels, as summarized in Table 4 and visualized in Fig 19.

**Table 4:** Load analysis statistic summary.

Users	Protocol	Response Time (ms)				Error %	Throughput (req/s)
		Average	Min	Max	Std. Dev.		
1	MQTT	119	86	954	23	0.0%	0.01
	Modbus	71	60	126	9	0.0%	0.01
	OPC UA	108	32	284	7	0.0%	0.02
	WebSocket	57	43	92	6	0.0%	0.02
5	MQTT	111	81	954	26	0.5%	0.03
	Modbus	145	86	201	15	0.0%	0.04
	OPC UA	126	32	390	13	0.0%	0.11
	WebSocket	61	40	115	9	0.0%	0.07
10	MQTT	107	80	2001	28	0.2%	0.03
	Modbus	262	140	394	26	0.0%	0.08
	OPC UA	140	30	422	14	0.0%	0.21
	WebSocket	68	42	301	13	0.0%	0.13
20	MQTT	106	79	2003	36	0.2%	0.04
	Modbus	501	159	589	30	0.0%	0.15
	OPC UA	176	30	487	16	0.0%	0.41
	WebSocket	83	44	152	17	0.0%	0.24
40	MQTT	113	78	2029	82	0.3%	0.04
	Modbus	951	310	2206	76	0.0%	0.26
	OPC UA	263	31	811	25	0.1%	0.77
	WebSocket	89	41	1096	31	0.0%	0.57
60	MQTT	119	78	2032	125	0.4%	0.04
	Modbus	1441	298	4089	156	0.0%	0.35
	OPC UA	332	31	978	31	0.0%	1.15
	WebSocket	96	42	1090	44	0.0%	0.47
80	MQTT	125	78	3044	139	0.4%	0.06
	Modbus	1885	1445	3784	133	0.0%	0.43
	OPC UA	394	32	1179	36	3.3%	1.47
	WebSocket	102	40	1134	40	0.0%	0.60

*Note:* The table provides summary statistics of the response times for each protocol under different user loads. The response time metrics include the average, minimum, maximum, and standard deviation values. Error percentage and throughput (requests per second) are also shown.

Among the protocols, WebSocket consistently achieved the lowest average response times across all user loads, starting at 57 ms for a single user and increasing modestly to 102 ms at 80 users. Minimum response times ranged narrowly from 40–43 ms, and the maximum response time reached 1,134 ms under the highest load. The standard deviation remained minimal, peaking at 44 ms for 60 users, indicating highly stable performance. WebSocket recorded zero errors (0.0%) up to 80 users and demonstrated the highest throughput, increasing from 0.02 to 0.60 requests/s as the load increased. These results confirm WebSocket’s efficiency, low latency, and scalability for real-time data transmission.

MQTT also performed well, with average response times increasing only slightly from 119 ms (1 user) to 125 ms (80 users), demonstrating robust latency stability under load. Minimum response times averaged around 78 ms, while maximum values ranged from 954 ms to 3,044 ms, suggesting potential instability during peak concurrency. The standard deviation rose from 23 ms to 139 ms with higher user loads, indicating moderate variability. MQTT maintained a low error rate, increasing marginally to 0.4% at 80 users, and achieved throughput growth from 0.01 to 0.06 requests/s. This performance demonstrates its suitability for moderate-scale IoT and control applications, where both low latency and reliability are essential.

OPC UA exhibited a progressive increase in average response time—from 108 ms at one user to 394 ms at 80 users—reflecting scalability limitations as the concurrent user load increased. Minimum response times averaged 32 ms, while maximum values grew from 284 ms to 1,179 ms, revealing moderate latency fluctuations. The standard deviation increased from 7 ms to 36 ms, consistent with the broader variability at higher loads. OPC UA maintained near-zero error rates at lower user levels, rising slightly to 3.3% at 80 users. Throughput increased from 0.02 to 1.47 requests/s, demonstrating strong scaling capability despite higher response times and minor reliability concerns at peak load.

Modbus displayed the highest latency among all protocols, with average response times increasing from 71 ms (1 user) to 1,885 ms (80 users). Minimum times started at 60 ms, while maximums expanded from 126 ms to 3,784 ms, indicating significant performance degradation under load. The standard deviation rose sharply from 9 ms to 133 ms, demonstrating high variability and reduced predictability. However, Modbus maintained a perfect 0.0% error rate across all user levels, confirming strong message integrity. Throughput increased from 0.01 to 0.43 requests/s, but its high latency and variability suggest limited suitability for time-critical applications.

Overall, WebSocket emerged as the most efficient protocol in terms of response time, stability, and throughput, making it ideal for real-time communication scenarios. MQTT offers balanced performance with moderate throughput and strong reliability, making it well-suited for IoT and telemetry systems. OPC UA scaled effectively in terms of throughput but exhibited increased response times and minor reliability issues under heavy loads, indicating potential scalability constraints. Modbus, while maintaining flawless delivery, suffered from the highest latency and jitter, making it less suitable for latency-sensitive industrial automation. Each protocol’s performance highlights trade-offs between scalability, responsiveness, and reliability, emphasizing the need for protocol selection based on application-specific requirements.

### 4.6. EC2 Node-RED server performance analysis

**Fig 20:**
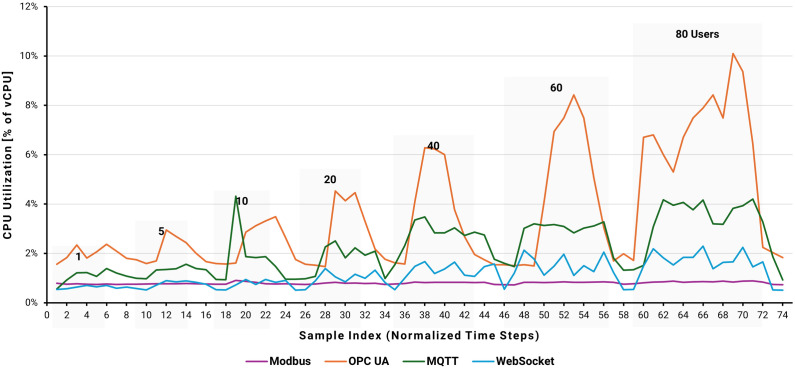
EC2 instance CPU utilization by user load.

**Fig 21:**
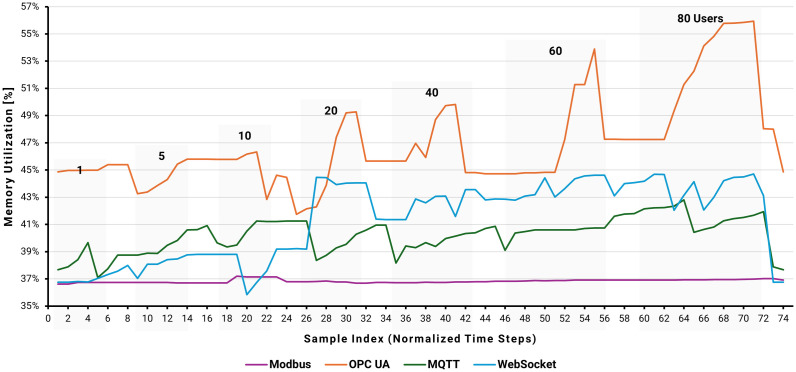
EC2 instance memory utilization by user load.

**Fig 22:**
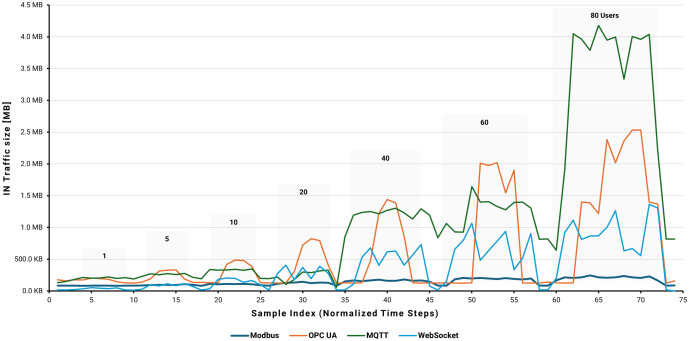
EC2 instance average network IN traffic by user load.

Figs 20–22 present a comparative analysis of the performance impact of Modbus, OPC UA, MQTT, and WebSocket protocols on an AWS EC2 instance running Node-RED under varying user loads simulated by JMeter. Metrics include CPU utilization, memory usage, and network traffic, providing a comprehensive view of system behavior under multi-user conditions.

In Fig 20, baseline CPU utilization remained below 1% with no JMeter traffic.

When a single-user load was initiated, OPC UA CPU usage rose sharply to approximately 2.5%, while other protocols remained at around 1%. As the number of users increased (5–80 users), OPC UA CPU utilization climbed steadily, peaking near 10% at 80 users. In contrast, Modbus consistently exhibited minimal CPU usage, remaining below 1% across all conditions. MQTT and WebSocket showed moderate increases, reaching approximately 4% and 2%, respectively, at 80 users. These results reflect OPC UA’s computational overhead due to complex data handling and metadata processing, while Modbus demonstrates minimal processing requirements.

Fig 21 illustrates memory utilization trends under identical load conditions. Without JMeter activity, baseline memory usage was approximately 37%. With increasing user loads, OPC UA memory consumption rose linearly—from roughly 38% with one user to 56% at 80 users—indicating substantial memory demand. Modbus again showed negligible change, maintaining around 37% usage regardless of load. MQTT and WebSocket exhibited moderate growth, increasing to approximately 41% and 44% at 20 users, then stabilizing as the load increased further. This pattern indicates that OPC UA’s memory usage scales with concurrency, while Modbus remains unaffected, and MQTT and WebSocket manage memory efficiently after an initial rise.

Network-in traffic, shown in Fig 22, further differentiates protocol behavior.

MQTT displayed the highest incoming network volume, peaking at approximately 4.2 MB at 80 users. OPC UA followed with 2.5 MB, reflecting frequent and complex data exchanges. WebSocket showed moderate traffic, reaching 1.4 MB at maximum load, while Modbus maintained the lowest network utilization, averaging about 0.3 MB. These results highlight MQTT’s higher network demands, OPC UA’s metadata-rich communications, WebSocket’s frequent small transmissions, and Modbus’s efficiency stemming from its compact message formats.

The integrated analysis of CPU, memory, and network utilization reveals distinct profiles of protocol-specific resource utilization. OPC UA is the most resource-intensive, with CPU usage peaking at nearly 10%, memory increasing linearly to 56%, and network traffic exceeding 2.5–3 MB at 80 users. Modbus is the most resource-efficient, with CPU usage below 1%, stable 37% memory utilization, and network traffic around 0.2–0.3 MB, making it ideal for resource-constrained environments. MQTT and WebSocket offer balanced performance, with CPU utilization reaching 2–4%, memory stabilizing around 43–45%, and network traffic between 2.0 and 4.0 MB. These characteristics make them suitable for scalable, cloud-based applications that require moderate resource overhead.

Performance differences arise from each protocol’s data-handling strategy. Modbus and MQTT efficiently pack multiple Boolean values—Modbus into a 16-bit word and MQTT into a byte array—minimizing message count and overhead. OPC UA, by contrast, writes each Boolean value individually as a separate operation, increasing processing and transmission load. WebSocket transmits individual Boolean values encapsulated in JSON objects, resulting in smaller but more frequent data packets. This variation in data packing and transmission frequency directly influences observed network and system utilization, underscoring the importance of selecting the appropriate protocol based on an application’s latency tolerance, network bandwidth, and computational constraints.

## 5. Discussion

### 5.1. Architectural advantages of the WebSocket–S7 bridge

The comprehensive analysis reveals a clear performance hierarchy among the evaluated protocols. The superior performance of the WebSocket–S7 bridge stems from its fundamental architecture: it establishes a full-duplex, persistent TCP connection that eliminates the overhead of repeated handshakes and broker intermediation (as in MQTT). This direct, low-overhead message framing is ideal for the high-frequency, small-payload data typical of PLC state changes. While OPC UA provides a rich information model and Modbus excels in simplicity, their query-based (polling and request–response) architectures inherently introduce latency—further exacerbated under wide-area network (WAN) conditions linking the cloud and local facilities.

The WebSocket–S7 bridge’s advantage lies in its hybrid design. The Web client handles lightweight WebSocket signaling, while Node-RED performs computationally intensive native S7 read/write operations on the server side, located close to the PLC. This separation of concerns—WebSocket for event-driven triggers and native S7 for deterministic control—removes the need to run a full industrial protocol stack across the WAN. By reducing serialization, handshake, and broker overhead, this hybrid model achieves the low latency and high stability observed in both local and remote experiments. These findings highlight that the latency improvements result not merely from transport tuning but also from deliberate architectural design that fuses web scalability with industrial determinism.

### 5.2. Protocol-specific performance analysis

To assess the influence of middleware and network topology on communication performance, each protocol was evaluated under two configurations: (i) a local setup using Node-RED hosted on the same LAN as the PLC, and (ii) a remote setup with Node-RED deployed on an AWS EC2 instance connected over a WAN. This dual testing isolates the additional latency introduced by cloud mediation, WAN propagation, and protocol handling in Node-RED’s runtime.

**Fig 23:**
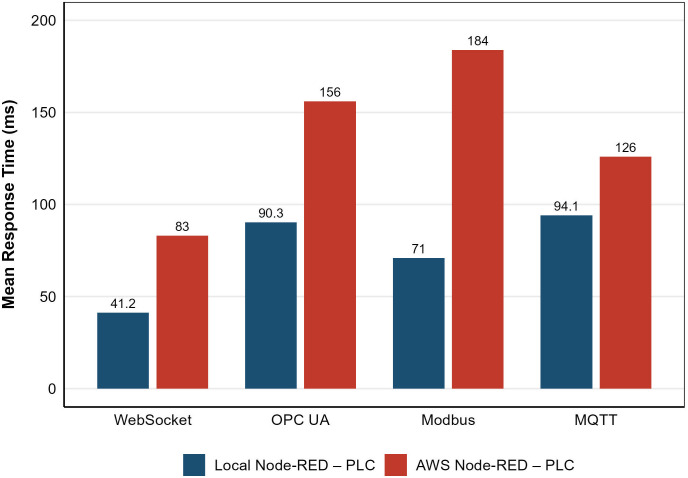
Comparison of mean RTT for each protocol in local and cloud setups.

Fig 23 summarizes the comparative performance of all tested protocols, while Table 5 provides the corresponding statistical breakdown of round-trip times (RTT). The local configuration establishes a baseline for middleware-mediated performance, whereas the AWS setup quantifies the cumulative impact of network distance, virtualization, and cloud scheduling variability.

**Table 5:** Comparison of local and AWS round-trip time (RTT) statistics and latency differencesΔ.

Statistic	Local (ms) [Node-RED]				AWS (ms) [Node-RED + Cloud]			
	WebSocket	OPC UA	Modbus	MQTT	WebSocket	OPC UA	Modbus	MQTT
Mean	41.2	90.3	71.0	94.1	83.0	156.0	184.0	126.0
Median	44.0	96.0	71.0	94.5	84.0	154.0	182.0	124.0
Minimum	10.0	7.0	27.0	36.0	30.0	28.0	45.0	55.0
Maximum	95.8	112.6	106.0	147.0	130.0	272.0	318.0	240.0
S.D.	16.2	28.6	15.4	18.0	18.0	35.0	42.0	38.0
Count	1083	1083	1083	1083	1083	1083	1083	1083
**Δ (AWS–Local)**	–	–	–	–	+41.8	+65.7	+113.0	+31.9
ANOVA (*p*-value)	***	***	***	***	***	***	***	***

*Note:* RTT was measured between the DTP application and the PLC. All results reflect communication through Node-RED, using a locally hosted instance for the local setup and a cloud-hosted instance for the AWS setup on a separate testing date. ΔRTTΔ represents the increase in average latency when transitioning from the local to the AWS Node-RED environment. The slight reduction in AWS RTT compared to previous tests reflects improved network stability while maintaining the same protocol performance hierarchy. A one-way ANOVA (*p* < 0.001) confirms statistically significant differences across all protocols under both setups.

Direct benchmarking was also conducted for Modbus TCP and OPC UA without Node-RED mediation. In these direct tests, Modbus achieved 10 ms RTT and OPC UA approximately 15 ms—values consistent with industrial Ethernet communication under ideal network conditions. However, once executed through Node-RED, RTTs increased to 71 ms and 90 ms respectively, primarily due to Node-RED’s event-loop overhead, JSON serialization, and scheduling delays. In contrast, WebSocket and MQTT inherently rely on middleware; thus, Node-RED naturally acts as their runtime environment and message broker.

WebSocket consistently outperformed other protocols, maintaining a sub-100 ms RTT even under cloud conditions and exhibiting the smallest increase between local and AWS environments (+41.8 ms). This superior performance is directly linked to several engineering optimizations implemented in WebSocket’s library. Persistent sessions eliminated repetitive TCP handshakes; compression was disabled to avoid CPU overhead; and the *TCP_NODELAY* flag prevented Nagle’s algorithm from aggregating small packets, thereby reducing jitter and maintaining deterministic cyclic timing. These design optimizations enabled WebSocket to maintain stable, low-jitter, full-duplex communication, closely aligned with the underlying network’s physical latency limits.

By contrast, MQTT exhibited moderate latency growth (+31.9 ms) due to broker processing and message queueing, but maintained excellent consistency thanks to its lightweight publish–subscribe structure. OPC UA showed a larger increase (+65.7 ms) due to its layered service model, metadata encoding, and subscription management overhead, while Modbus presented the most substantial latency increase (+113 ms).

The high latency in Modbus is primarily due to its 80 ms polling rate between read and write cycles in the nodes—necessary to balance responsiveness and CPU utilization within Node-RED. Attempts to reduce this interval below 80 ms resulted in message congestion and delayed responses, as the Node-RED engine struggled with the increased message frequency. Moreover, Modbus’s inherently sequential request–response pattern forces each transaction to await acknowledgment before the next begins, amplifying total latency under load. These cumulative factors led to perceptible sluggishness in the virtual model’s operation and occasionally caused the machine cycles to fail to synchronize in real time. Such limitations may be mitigated by optimizing polling intervals, employing asynchronous Modbus handlers, or deploying Node-RED flows on multi-threaded runtimes.

During initial OPC UA testing, round-trip times frequently exceeded 500 ms until the *minimumSamplingInterval* parameter in the official OPC UA Server Node (104-opcuaserver.js) was reduced from 500 ms to 50 ms. This adjustment enhanced variable sampling frequency and responsiveness, underscoring the critical role of parameter tuning in aligning OPC UA with real-time applications. Alternative configurations with the PLC acting as both Modbus and OPC UA clients were also tested; however, they required port forwarding to the remote PLC and introduced additional latency and configuration complexity compared to the Node-RED-mediated approach. In contrast, MQTT required no port forwarding and maintained stable timing, reaffirming its efficiency for distributed, event-driven applications.

Overall, these findings demonstrate that WebSocket–S7 achieves the most balanced trade-off between low latency, resource efficiency, and scalability. Its optimized event-driven architecture, coupled with transport-level enhancements, allows it to match the responsiveness of direct industrial protocols even under cloud-based conditions—preserving deterministic timing essential for real-time digital twin synchronization.

### 5.3. System resource and scalability trade-offs

Node-RED monitoring revealed that the WebSocket–S7 approach outperformed all others in latency, reliability, and scalability, achieving an average round-trip time of 87 ms—well below the IEC 61588 threshold of 100 ms for real-time operations and 200 ms for simulated processes. Memory utilization for WebSocket increased with the number of concurrent users, peaking at 44% for 20 users. MQTT demonstrated balanced performance, with an average latency of 133 ms and memory stabilization at 41%. OPC UA and Modbus achieved average latencies of 162 ms and 194 ms, respectively, meeting the requirements for simulated processes but requiring optimization for real-time use. Modbus consumed the least resources but scaled poorly, while OPC UA handled complex data robustly at the cost of higher memory usage (up to 56%).

These findings suggest that WebSocket is well-suited for high-frequency, feedback-driven digital twins, whereas MQTT, OPC UA, and Modbus are more suitable for simulated or slower control processes. Table 6 summarizes these key trade-offs. WebSocket–S7 offers real-time responsiveness and scalability; MQTT provides modular decoupling between producers and consumers; OPC UA suits metadata-rich systems; and Modbus remains valuable for simple or legacy integrations where low resource use is paramount.

**Table 6:** Protocol comparison and trade-offs.

Protocol	Avg. Latency (ms)	Server Load	Scalability	Key Advantage	Key Disadvantage
**WebSocket–S7**	**87**	Low (CPU/Mem)	**Excellent**	**Lowest latency; high scalability**	Requires custom bridge logic; less standardized implementation
MQTT	133	Moderate	Good	Standardized pub/sub; decoupled architecture	Broker adds latency and a single point of failure
OPC UA	162	**High**	Moderate	Rich, standardized data model; built-in security	High server resource usage; complex configuration
Modbus TCP	194	**Very Low**	Poor	Extreme simplicity; universal support	**Highest latency; poor scalability**; no built-in security

*Note:* Bold values highlight the best-performing attributes, including low latency, low server load, and strong scalability, as well as negative extremes, including the highest latency, highest load, and poor scalability.

### 5.4. Architectural roots of performance differences

Additional WebSocket optimizations—persistent sessions, disabled compression, and *TCP_NODELAY*—further reduced serialization overhead and reinforced its latency advantage. The performance hierarchy observed originates from fundamental architectural design rather than implementation details.

WebSocket maintained stable latency because its persistent full-duplex TCP session avoids repeated handshakes and broker queuing, enabling event-driven transmission ideal for frequent, small PLC updates. MQTT’s broker-mediated routing introduced moderate queuing delay under concurrency. OPC UA’s semantic richness and layered services added CPU and memory overhead due to metadata serialization and subscription management. Modbus TCP’s sequential request–response model introduced head-of-line blocking and compounded polling delays.

Thus, the observed hierarchy is governed more by connection management and data model complexity than by raw bandwidth. WebSocket amortizes connection setup costs, achieving the lowest latency (87 ms); MQTT balances scalability and latency; OPC UA trades performance for interoperability; and Modbus prioritizes simplicity at the cost of responsiveness.

**Optimization Rationale in Industrial Contexts.** Each WebSocket enhancement directly addressed industrial communication constraints. Disabling Nagle’s algorithm using *TCP_NODELAY* prevented packet coalescence delays that would otherwise disrupt deterministic PLC scan cycles. Persistent sessions removed repetitive TCP handshakes, maintaining continuity across control iterations. Variable batching, which mirrors PLC cyclic data grouping, reduces message frequency without losing event granularity, while disabling compression avoided CPU overhead on constrained field devices. Collectively, these optimizations align WebSocket behavior with industrial timing needs—low jitter, bounded latency, and deterministic cyclic updates essential for real-time PLC–digital twin synchronization.

### 5.5. Implications for engineering education

Although this study focused on technical performance, the demonstrated sub-100 ms response times have direct educational relevance. The WebSocket–S7 bridge sustained consistent performance with up to 40 concurrent users (Section 4.5), meeting the requirements for classroom-scale digital twin deployments where multiple students interact with virtual equipment simultaneously. This enables realistic, responsive training environments that mirror industrial operations, fostering situational awareness and troubleshooting skills. The framework’s low client-side demands and web-based accessibility further support deployment using standard student devices.

These findings align with prior work on digital twin–based education [17,24,26,49], where low-latency, real-time interaction enhances engagement and conceptual understanding [27,50]. The demonstrated scalability and deterministic timing make this framework a strong foundation for remote and hybrid PLC laboratories consistent with Industry 5.0’s human-centric learning goals. Future studies should evaluate learning outcomes, engagement, and skill transfer compared to conventional labs.

### 5.6. Generalizability and platform considerations

Although this study validated the proposed WebSocket–S7 bridge using a single PLC model (Siemens S7-1500) and a single cloud environment (AWS EC2), these choices ensured a controlled and reproducible testbed aligned with industry-grade hardware and widely used cloud infrastructure. The findings, therefore, represent a verified proof of concept rather than a vendor-specific optimization. The framework itself is platform-agnostic: the WebSocket layer functions as an intermediate, event-driven trigger that can operate alongside existing industrial protocols. It can be adapted to other PLC brands (e.g., Allen-Bradley, Beckhoff) or cloud providers (e.g., Azure, Google Cloud) with minimal modification to the Node-RED configuration. Future work will extend testing across multiple PLC brands and architectures, as well as deployments spanning different cloud regions and hybrid edge–cloud environments, to further evaluate scalability and interoperability in heterogeneous industrial contexts.

### 5.7. Measurement uncertainty and network effects

While every effort was made to ensure measurement accuracy, several potential sources of uncertainty should be acknowledged. Network jitter in the inter-city WAN path between Al Ain and Abu Dhabi could introduce variability in round-trip times. To mitigate this, all protocol tests were conducted during consistent time intervals (the same hours across multiple days), with preliminary ICMP validation showing a stable baseline latency of 18–21 ms before each test session. Each measurement condition was repeated across 2,200 interactions per protocol to ensure statistical significance. Clock synchronization between the Android device, AWS instances, and PLC introduced minor timing discrepancies, which were mitigated through the use of a high-precision *System.Diagnostics.Stopwatch* in Unity and the TIA Portal’s Long-Term Trace tool. Background processes on the Android device and EC2 instances may have contributed to occasional latency spikes observed as outliers in the results. Additionally, the 150 km geographical separation inherently introduces a propagation delay that is factored into all measurements. These factors collectively contribute to the observed standard deviations but do not alter the fundamental performance hierarchy among protocols.

### 5.8. Limitations and future work

Although the purpose of this study was to achieve optimal round-trip times by simulating a real machine process, the study has several limitations related to industrial environments. First, the experiment was conducted in a controlled environment using Boolean variables without considering different types and sizes of variables, which may not fully reflect the complexities and variability of real-world industrial settings. Second, the scope was limited to specific protocols, and the findings may not apply to other communication protocols or configurations. Third, the hardware and network conditions in this experiment may differ from those in different industrial applications, potentially affecting the reproducibility of the results. Lastly, the focus was mainly on performance metrics, without addressing other crucial factors such as security and cost. Future research will also include pilot testing in live industrial environments to validate system robustness under operational workloads and multi-region cloud deployments.

The system included reconnection and session-recovery mechanisms (Section 3.2.2) but did not undergo systematic fault injection. Scenarios such as sustained packet loss, network partitions, or hardware-induced link failures were not experimentally evaluated. Consequently, while the framework demonstrated bounded recovery under incidental WAN fluctuations, a comprehensive assessment of fault tolerance, recovery time objectives, and degraded-mode behavior remains necessary for production-grade deployment and will form part of future research.

Security considerations also warrant further study. Although WebSocket (WSS), MQTT (MQTTS), and OPC UA natively support TLS/SSL encryption, and Modbus typically relies on network-level security (e.g., VPNs), the performance overhead introduced by these mechanisms has not been quantified. Future research should measure the latency and CPU costs of secure implementations, as this trade-off is crucial for the industrial adoption of secure systems.

## 6. Conclusion

This study demonstrates that the selection of a communication protocol critically influences the responsiveness and scalability of cloud-based digital twins. The proposed WebSocket–S7 bridge achieved the lowest average latency (87 ms), highest scalability under load, and minimal client resource consumption, meeting the IEC 61588 real-time threshold. Its lightweight, event-driven architecture enables direct, reliable synchronization between cloud applications and industrial controllers, supporting both educational and industrial use cases that demand rapid feedback and high-fidelity process visualization.

For educational settings, the framework provides realistic, responsive remote laboratory environments that align with the human-centric goals of Industry 5.0, enabling safe, accessible, and interactive training. MQTT remains a viable alternative for moderately time-sensitive applications, while OPC UA and Modbus provide strong interoperability for complex or legacy systems, albeit with higher overhead. Collectively, these findings establish a benchmark for selecting communication strategies in digital twin implementations.

Future work will explore three main directions: integrating encryption mechanisms to evaluate the security–performance trade-off; extending the analysis to diverse data types and industrial protocols such as CoAP and DDS; and deploying hybrid edge–cloud architectures, including 5G-enabled environments, to further reduce latency and enhance system resilience. These next steps aim to generalize the proposed architecture for broader industrial and educational deployment.

## Supporting information

S1 Table Technical hardware specifications for the experimental setup. (PDF)

S2 Table Virtual component/PLC inputs and outputs. (PDF)

S1 Fig AWS Node-RED flow with an OPC UA server node. (TIF)

S2 Fig Local Node-RED flow with an OPC UA client node. (TIF)

S3 Fig Local Node-RED flow with OPC UA client node—read from server. (TIF)

S4 Fig AWS Node-RED flow with a Modbus server node—read from PLC. (TIF)

S5 Fig Local Node-RED flow with a Modbus client node. (TIF)

S6 Fig PLC ladder logic program for the automated box-sorting plant. (TIF)
