# Supplementary material for: Toward Industry 5.0: A WebSocket–S7 Bridge for Low-Latency, IEC 61588-Compliant Digital Twins in Remote Industrial Automation
Source: PLoS One. 2026 May 11;21(5):e0342004. doi: 10.1371/journal.pone.0342004 (PMC13160324; doi:10.1371/journal.pone.0342004)
Supplement: S1 Table — (PDF) [file pone.0342004.s001.pdf]

S1 Table. Hardware technical specifications for the experimental setup.

Device	Lenovo K5	Computer	AWS EC2	Siemens PLC
**Model**	Lenovo L38011	DESKTOP	t3.micro	S7-1500
**CPU**	Qualcomm Snapdragon 430 (1.4 GHz)	12th Gen Intel i5-12400F (2.5 GHz, 6 cores)	Intel Xeon Platinum 8259CL (2.50 GHz)	CPU 1517TF-3 PN/DP
**RAM**	3 GB	48 GB	1 GB	3 MB
**Storage**	32 GB	500 GB SSD	8 GB	8 MB
**System**	Android v8.0.0	Windows 11 Pro (64-bit)	Linux 2023 AMI x86_64 kernel-6	Firmware V2.9
**W/NIC**	Wi-Fi 802.11 a/b/g/n (450 Mbps)	Realtek PCIe GbE (2.5 GB/s)	Elastic Network Adapter (ENA)	PROFINET interfaces (TCP/IP)
