# Supplementary material for: Toward Industry 5.0: A WebSocket–S7 Bridge for Low-Latency, IEC 61588-Compliant Digital Twins in Remote Industrial Automation
Source: PLoS One. 2026 May 11;21(5):e0342004. doi: 10.1371/journal.pone.0342004 (PMC13160324; doi:10.1371/journal.pone.0342004)
Supplement: S2 Table — (PDF) [file pone.0342004.s002.pdf]

S2 Table. Virtual component/PLC inputs and outputs.

PLC Addresses	Type	Virtual Components	Description
DB1.DBX0.0	Input	Start Push Button	Starts the machine
DB1.DBX0.1	Input	Stop Push Button	Stops the machine
DB1.DBX0.2	Input	InfraRed Sensor 1	Package presence detection
DB1.DBX0.3	Input	InfraRed Sensor 2	Package presence detection
DB1.DBX0.4	Input	InfraRed Sensor 3	Package presence detection
DB1.DBX0.6	Input	Package Scanner Sensor	Damaged package detection
DB1.DBX0.7	Input	Actuator 2 ON_Sensor	Piston 2 opening position
DB1.DBX1.0	Input	Actuator 2 OFF_Sensor	Piston 2 closing position
DB1.DBX1.1	Input	Actuator 1 ON_Sensor	Piston 1 opening position
DB1.DBX1.2	Input	Actuator 1 OFF_Sensor	Piston 1 closing position
DB1.DBX1.3	Output	Belt 1 Actuator	Moving the packages
DB1.DBX1.4	Output	Belt 2 Actuator	Moving the packages
DB1.DBX1.5	Output	Actuator 1 Forward	Piston 1 forwarding
DB1.DBX1.6	Output	Actuator 1 Reverse	Piston 1 reversing
DB1.DBX1.7	Output	Actuator 2 Forward	Piston 2 forwarding
DB1.DBX2.0	Output	Actuator 2 Reverse	Piston 2 reversing
DB1.DBX2.1	Output	Pusher 3 Forward	Piston 3 forwarding
DB1.DBX2.5	Output	Scanner Red Lamp	Damaged package indicator
DB1.DBX2.6	Output	Scanner Green Lamp	Machine running indicator
