# Supplementary figures and images for: Toward Industry 5.0: A WebSocket–S7 Bridge for Low-Latency, IEC 61588-Compliant Digital Twins in Remote Industrial Automation
Source: PLoS One. 2026 May 11;21(5):e0342004. doi: 10.1371/journal.pone.0342004 (PMC13160324; doi:10.1371/journal.pone.0342004)

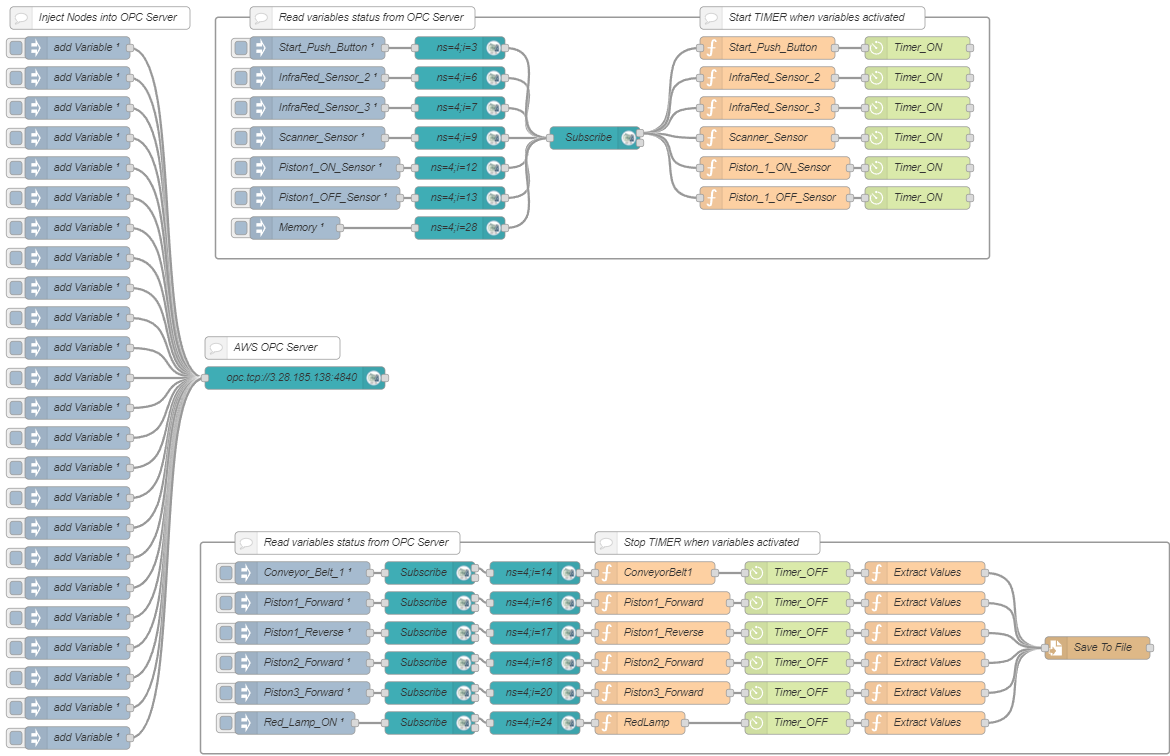

Supplement: S1 Fig — (TIF) [file pone.0342004.s003.tif]

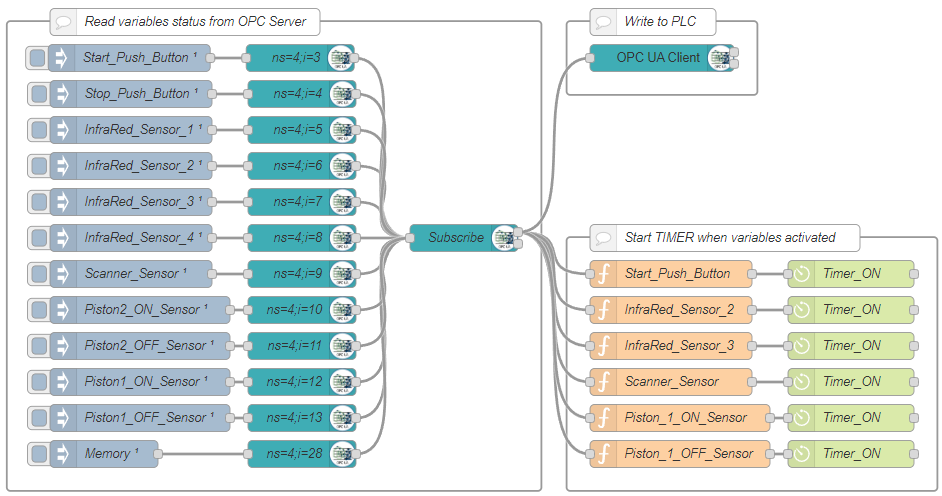

Supplement: S2 Fig — (TIF) [file pone.0342004.s004.tif]

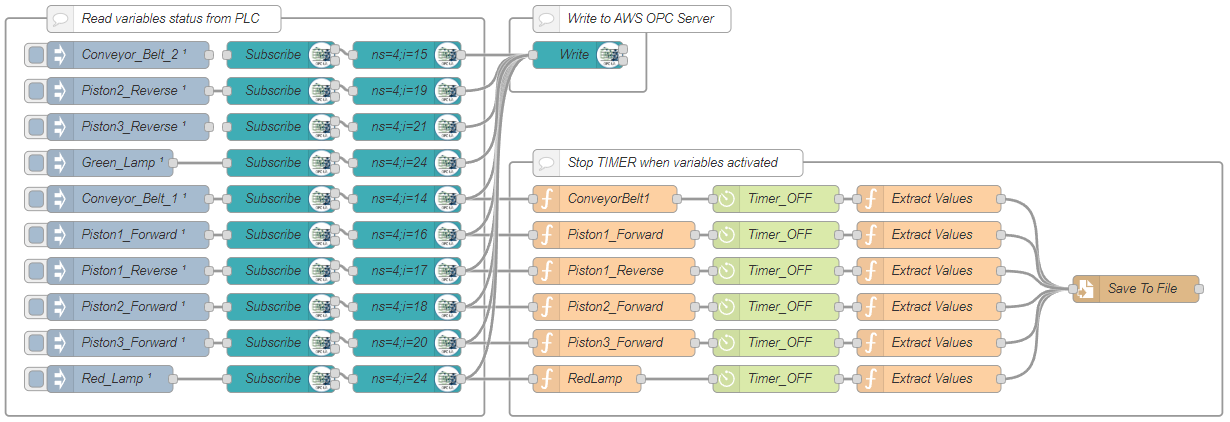

Supplement: S3 Fig — (TIF) [file pone.0342004.s005.tif]

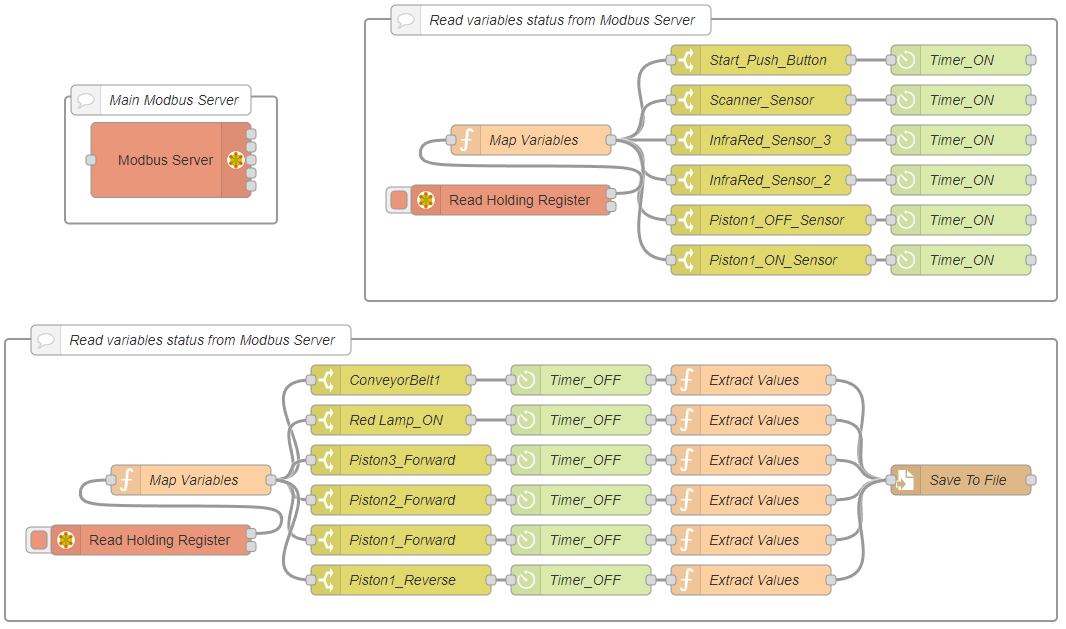

Supplement: S4 Fig — (TIF) [file pone.0342004.s006.tif]

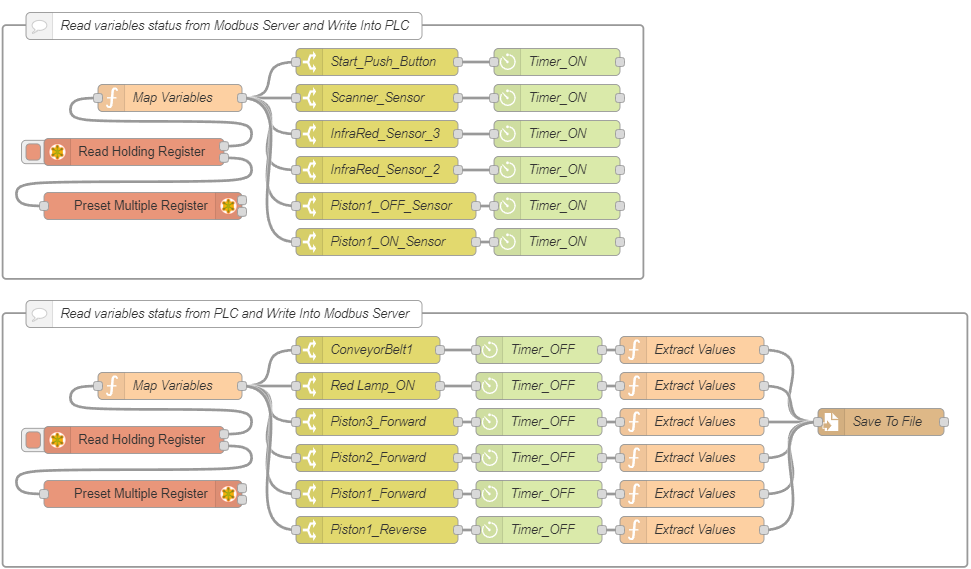

Supplement: S5 Fig — (TIF) [file pone.0342004.s007.tif]

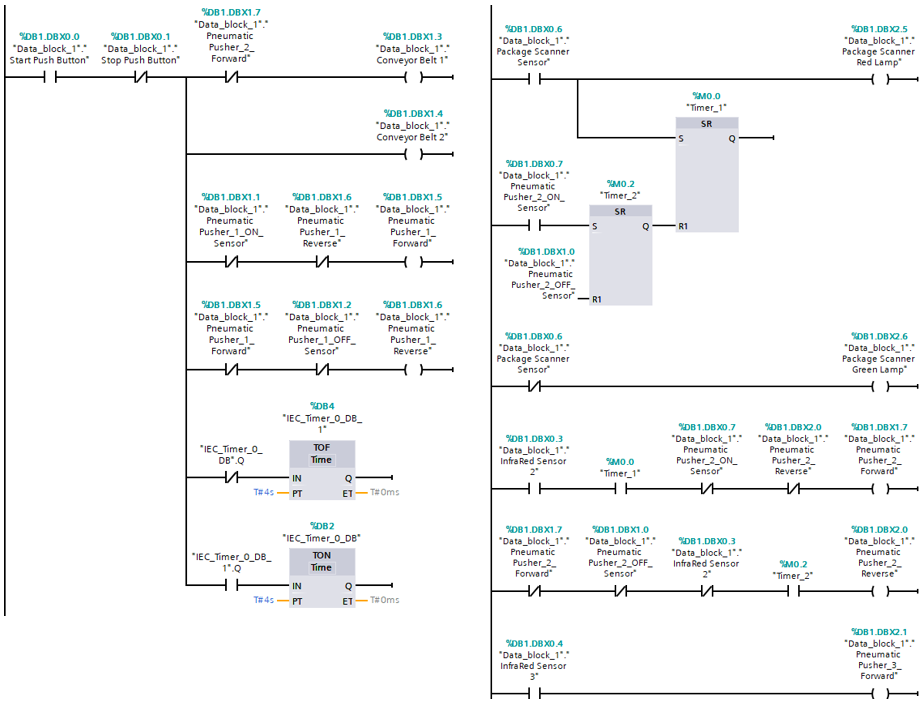

Supplement: S6 Fig — (TIF) [file pone.0342004.s008.tif]
